# A novel antibacterial and antifouling nanocomposite coated endotracheal tube to prevent ventilator-associated pneumonia

**DOI:** 10.1186/s12951-022-01323-x

**Published:** 2022-03-05

**Authors:** Yue Wang, Bingyue Cai, Dalong Ni, Yu Sun, Gang Wang, Hong Jiang

**Affiliations:** 1 Department of Anesthesiology, Shanghai Ninth People’s Hospital, Shanghai Jiao Tong University School of Medicine, No. 639 Zhizaoju Rd, Shanghai, 200011 People’s Republic of China; 2 State Key Laboratory for Modification of Chemical Fibers and Polymer Materials, College of Materials Science & Engineering, Donghua University, No. 2999 Renmin Rd, Shanghai, 201620 People’s Republic of China; 3 Department of Orthopaedics, Shanghai Key Laboratory for Prevention and Treatment of Bone and Joint Diseases, Shanghai Institute of Traumatology and Orthopaedics, Ruijin Hospital, Shanghai Jiao Tong University School of Medicine, No. 197 Ruijin 2nd Rd, Shanghai, 200025 People’s Republic of China

**Keywords:** Chitosan-nano silver, Surface modification, Ventilator-associated pneumonia, Antibacterial, Antifouling

## Abstract

**Background:**

The endotracheal tube (ETT) is an essential medical device to secure the airway patency in patients undergoing mechanical ventilation or general anesthesia. However, long-term intubation eventually leads to complete occlusion, ETTs potentiate biofilm-related infections, such as ventilator-associated pneumonia. ETTs are mainly composed of medical polyvinyl chloride (PVC), which adheres to microorganisms to form biofilms. Thus, a simple and efficient method was developed to fabricate CS-AgNPs@PAAm-Gelatin nanocomposite coating to achieve dual antibacterial and antifouling effects.

**Results:**

The PAAm-Gelatin (PAAm = polyacrylamide) molecular chain gel has an interpenetrating network with a good hydrophilicity and formed strong covalent bonds with PVC-ETTs, wherein silver nanoparticles were used as antibacterial agents. The CS-AgNPs@PAAm-Gelatin coating showed great resistance and antibacterial effects against *Staphylococcus aureus* and *Pseudomonas aeruginosa*. Its antifouling ability was tested using cell, protein, and platelet adhesion assays. Additionally, both properties were comprehensively evaluated using an artificial broncho-lung model in vitro and a porcine mechanical ventilation model in vivo. These remarkable results were further confirmed that the CS-AgNPs@PAAm-Gelatin coating exhibited an excellent antibacterial capacity, an excellent stain resistance, and a good biocompatibility.

**Conclusions:**

The CS-AgNPs@PAAm-Gelatin nanocomposite coating effectively prevents the occlusion and biofilm-related infection of PVC-ETTs by enhancing the antibacterial and antifouling properties, and so has great potential for future clinical applications.

**Graphical Abstract:**

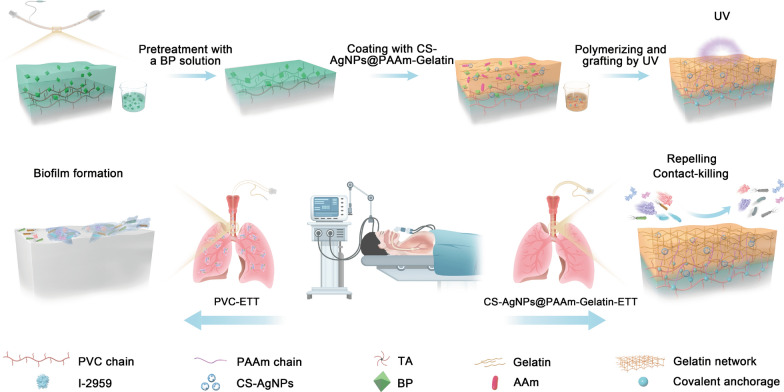

**Supplementary Information:**

The online version contains supplementary material available at 10.1186/s12951-022-01323-x.

## Introduction

In clinical practice, mechanical ventilation with endotracheal tubes (ETTs) is commonly employed in operations carried out under general anesthesia and in the treatment of critically ill patients. Tracheal intubation using ETTs is one of the most reliable means of maintaining airway patency, and it is reported that annually, 50 million patients worldwide have been treated by tracheal intubation using ETTs to achieve respiratory assistance [1]. Unfortunately, although regarded as life-saving equipment, ETTs also frequently pose a threat to the patient, since they can undergo occlusion by mucus and secretions, in addition to being coated with biofilms. More specifically, long-term ETT intubation leads to the accumulation of mucus and secretions, a reduction in the airway patency, and eventually, complete occlusion [2, 3]. Indeed, this process occurs to varying degrees in all intubated patients, beginning within hours of endotracheal intubation [4]. As one example, mechanical ventilation with tracheal intubation has become essential in the management of severe COVID-19 infections [5], and in a recent study, a high ETT occlusion rate (i.e., ≤ 72%) has been described in COVID-19 patients [6]. In this context, a microscopic examination of a patient’s ETT content showed a trilaminar appearance, with a superficial layer of mucin, inflammatory cell degeneration, and intercellular stroma [7]. In addition, an autopsy report confirmed that a large amount of mucus was found in the lungs of COVID-19 patients, which blocked the alveoli, and resulted in an impaired lung function [8]. It is also known that ETTs potentiate biofilm-related infections, such as ventilator-associated pneumonia (VAP). More specifically, intubated patients are prone to VAP because the hydrophobic surface of the polyvinyl chloride (PVC)-ETT provides ideal conditions for pathogen attachment and grow, ultimately resulting in bacterial coverage over the entire internal surface [9–11]. These biofilms become a persistent source of pathogenic bacteria that can spread to the lower airways, invading the lungs, and causing VAP.

Current designs of ETT surface modifications include active or passive methods and combinational approaches that address biomaterial development [12, 13]. As an active method to kill microbes on device surfaces, the ETT can be coated with various antiseptic substances to prevent VAP. Examples of such antiseptic substances include silver nanoparticles (AgNPs) [14, 15], silver sulfadiazine/chlorhexidine [16], zinc oxide [17], antimicrobial peptide [18], and photodynamic therapy [19, 20] wherein the incorporation of these substances into surface coatings has been found to minimize biofilm formation and bacterial growth. In addition, some passive methods to deal with microbial adhesion have been applied to ETTs to produce a contamination-resistant surface by altering the surface composition and pattern. For example, photolithographic etching of the Sharklet™ micro-pattern onto a silicone ETT surface resulted in a significant decrease in the accumulation of mucus inside the ETT, both in vitro and in vivo [21]. In addition, nanorough ETTs that can decrease bacterial growth and biofilm formation have also been introduced [22]; however, their widespread use is hindered by concerns over antibiotic resistance, coating stabilities, biocompatibility, and relatively high costs. Indeed, only silver-coated ETTs have been approved as antibiofilm ETTs in the USA, and to date, these coatings have been subjected to multiple clinical trials [14]. Although the silver-coating of ETTs resulted in effective antibacterial activities, they depend on the release of Ag^+^ ions from silver salts, which has raised concerns related to cytotoxicity and stability.

**Scheme 1:**
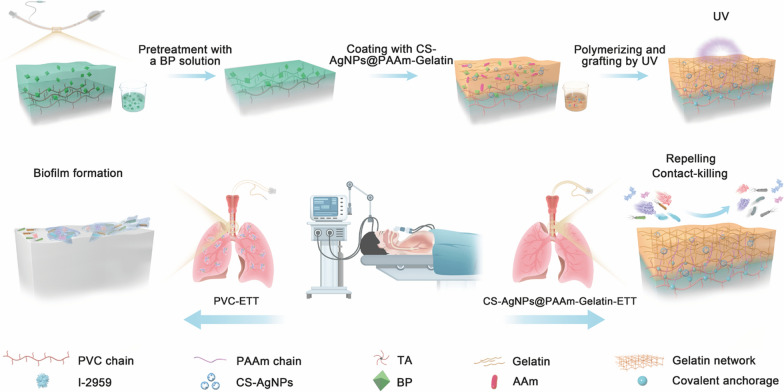
Schematic representation of the CS-AgNps@PAAm-Gelatin-ETT assembly process and its antibacterial and antifouling mechanism. PVC substrate was pretreated with benzophenone (BP), and then PAAm and gelatin were employed to form an interpenetrating network structure, in which the CS-AgNPs nanocomposites were uniformly distributed to achieve dual antibacterial and antifouling effects, and effectively prevent the occlusion of traditional PVC-ETTs and the incidence of VAP

It was therefore considered that a superior effect could be achieved by combining both active and passive strategies for the surface modification of ETTs. Thus, as shown in Scheme 1, we herein report the development of a nanocomposite of CS-AgNPs@PAAm-Gelatin coating on the ETT surface, which endows the ETT with excellent active antibacterial and passive antifouling properties. As a natural biopolymer derived from the deacetylation of chitin, chitosan (CS) biomaterials possess a wide range of antibacterial and antifungal activities with many active amino groups, which can provide active sites for complexation with metal ions [23]. For example, AgNPs can be combined with chitosan to increase their antibacterial activities [24–26]. In addition, the PAAm-Gelatin (PAAm = polyacrylamide) molecular chain gel has an interpenetrating network with a good hydrophilicity, and can effectively prevent microbial aggregation and adhesion. Thus, in this study, silver ions are sequentially complexed on the surface of chitosan prior to their reduction to AgNPs (giving CS-AgNPs) and their introduction into the PAAm-Gelatin network. The surface of an ETT is then coated with the obtained CS-AgNPs@PAAm-Gelatin, in which the covalent bond between the gelatin and the ETT substrate is formed under ultraviolet irradiation. The CS-AgNPs@PAAm-Gelatin coating is evaluated in vitro to examine its influence on mucus build-up, bacterial adhesion, biofilm formation, and airway narrowing in the ETT, and a preliminary toxicity study is carried out. Finally, the in vivo therapeutic outcomes are examined to determine the ability of the CS-AgNPs@PAAm-Gelatin-ETT system to prevent VAP and the occlusion of traditional PVC-ETTs in a porcine mechanical ventilation model.

## Materials and methods

### Materials

All agents were obtained commercially as follows: Acrylamide (AM, purity 99%,Aladdin Chemical Co., Ltd.), 2-hydroxy-4′-(2-hydroxyethoxy)-2-methylpropiophenone (I-2959, purity 98%, Sigma-Aldrich), tannic acid (TA, Mr = 1701.2, Aladdin Chemical Co., Ltd.), gelatin (~ 250 g Bloom, Aladdin Chemical Co., Ltd.), BP (purity 99%, Aladdin Chemical Co., Ltd.), CS (DD = 90%, Shanghai Yuanye Biology), silver nitrate (AgNO_3_, purity 99%, SCRC), sodium borohydride (NaBH_4_, purity 99%, Aladdin Chemical Co., Ltd.), acetic acid (HAc, purity 99%, Merck), acetone (purity 99%, SCRC), and ethanol (EtOH, purity 99%, SCRC). The planar PVC was purchased from a Shanghai commodity store. All chemicals were used without further purification. Deionized water (Milli-Q system) was used throughout the experiments.

### Preparation of the CS-AgNps@PAAm-Gelatin nanocomposite coating

Scheme 1 schematically illustrates the procedure used for fabrication of the CS-AgNPs@PAAm-Gelatin coating. As the main component of ETTs, PVC substrate was coated with CS-AgNPs@PAAm-Gelatin for the purpose of this study. Initially, the CS-AgNPs antibacterial agent was prepared by dissolving CS (1% w/v) in an aqueous HAc solution (1% v/v). Subsequently, a AgNO_3_ solution (15 mL, 10, 20, or 40 mM) was mixed with the CS solution (12.3 mL), and a NaBH_4_ solution (2.7 mL, 112, 224, or 448 mM) was added slowly to the CS solutions containing different AgNO_3_ concentrations. A solution containing the hydrophilic monomers was then prepared, wherein AAm (20 wt.%), I-2959 (1 wt.%), gelatin (27 wt.%), and TA(3.5wt.%) were mixed in water at 85 ℃, and the obtained CS-AgNPs solution (1% v/v) was added and mixed. Subsequently, PVC was dipped into a BP solution (10 wt.%) in EtOH/acetone (1:4, v/v) for 5 min, then dried under a stream of N_2_ prior to coating with the pre-solution, and treatment with ultraviolet irradiation in an ultraviolet chamber (365 nm ultraviolet; UVP CL-1000) for 1 h to produce the desired CS-AgNps@PAAm-Gelatin nanocomposite coating. The PAAm-Gelatin coating was prepared in the same manner but without addition of the CS-AgNPs solution.

### Morphology and component characterization

The UV–visible (UV–Vis) spectra were recorded using a UV-3200 UV–Vis spectrometer (Shanghai, China), while the Fourier transform infrared (FTIR) spectra were recorded using a Nicolet 6700 FTIR spectrometer (Thermo Fisher Scientific, USA). The morphologies of the samples were evaluated by scanning electron microscopy (SEM, S-4800, Japan), and Image J software (National Institute of Health, USA) was used to quantify the thickness of coatings. X-ray photoelectron spectroscopy (XPS) data were documented with AXIS Ultra DLD (Kratos, British). X-ray diffraction (XRD) patterns were recorded using an AXIS Ultra DLD (Kratos, UK), while the X-ray diffraction (XRD) patterns were recorded using a Bruker d8 Advance X-ray diffractometer with Cu Kα radiation (λ = 1.5406 Å) at an acceleration voltage of 40 kV, and at 40 mA in the θ − 2θ scan mode. The water contact angle (WCA) measurements were carried out using a ThetaFlex instrument (Biolin Scientific, Finland). The atomic force microscopy (AFM) images were obtained using a Bruker Dimension Icon AFM (USA), while atomic absorption spectrometry (AAS) was performed with a SpectrAA 220FS instrument (VARIAN, USA) to detect the compositions of the released solutions and the total Ag^+^ contents of the coatings. A Q-Sense E4 quartz crystal microbalance with dissipation monitoring (QCM-D) (Q-Sense AB, Västra Frölunda, Sweden) equipped with a gold-plated quartz crystal (5 MHz AT-cut) (QSX 301, Q-Sense AB, Västra Frölunda, Sweden) was used to estimate the dynamic adsorption of bovine serum albumin (BSA), human serum albumin (HSA), and cytochrome c. The fluorescence intensity of adsorbed proteins was measured by microplate reader (Synergy H1, USA). The bacterial and cell activities were determined by confocal laser scanning microscopy (CLSM) (Leica, Germany). To determine whether surface modification compromises the mechanical properties of PVC, including its fracture strength, both PVC-ETTs and CS-AgNPs@PAAm-Gelatin-ETTs were examined, and the relevant tests were performed by the universal mechanical tester.

### In vitro antibacterial tests

#### Bacteria preparation

*Staphylococcus aureus* (*S. aureus*, ATCC 29213) and *Pseudomonas. aeruginosa* (*P. aeruginosa*, ATCC 15442) were used to detect the antibacterial capabilities of the samples through in vitro experiments. Both bacteria were cultivated in tryptic soy broth (TSB) medium at 37 °C and diluted with phosphate-buffered saline (PBS).

#### Agar diffusion assay

An agar diffusion assay was performed to determine the antibacterial capacities of the different samples. The bacteria were pre-cultured and diluted to 1 × 10^7^ colony-forming units (CFU) mL^−1^ with TSB. An aliquot (200 μL) of the diluted bacterial suspension was then evenly spread onto the agar surface. Samples were divided into six groups: Blank, PVC, CS (40 ppm) and different concentrations of CS-AgNPs (i.e., Ag50, Ag100, and Ag200 ppm) and gently placed onto the center of the agar surfaces and cultured at 37 °C for 18 h. After this time, the antibacterial capacity was evaluated by observing the width of the inhibition zones surrounding the samples.

#### Bacterial counting assay

The bacterial counting assay was used to evaluate the antibacterial activity of each sample. A bacterial suspension (100 μL) containing 1 × 10^8^ CFU mL^−1^ bacteria was dropped onto PVC, CS@PAAm-Gelatin-PVC and CS-AgNps@PAAm-Gelatin-PVC substrates using PBS as control. After culturing for 24 h at 37 °C, the samples were transferred into sterilized centrifuge tubes containing PBS (4 mL). The samples were then agitated intensely for 30 s to detach the bacteria from the sample surfaces. Subsequently, the obtained suspension containing the detached-bacteria was diluted 1000 times with PBS. An aliquot (10 μL) was spread onto a standard agar plate and cultured at 37 °C for another 18 h to image the surviving bacterial colony. An aliquot (100 μL) was evenly spread onto SBA plates and cultured at 37 °C for 18 h to count the surviving bacterial colonies. The antibacterial percentage was calculated using the following formula:

$${\text{P }}\left( \% \right) = \left( {{\text{A}} - {\text{B}}} \right) \div {\text{A}} \times 100\% ,$$

where A represents the average number of bacterial colonies in the blank, and B represents the average number of bacterial colonies of the different samples.

#### Bacterial morphology

SEM was used to examine the morphology of the bacteria adhering to the sample surfaces. For this purpose, bacterial suspensions (100 μL) containing 1 × 10^7^ CFU mL^−1^ of bacteria were dropped onto the sample surfaces. After 6 h of culture at 37 °C, the samples were washed gently with PBS and fixed with 2.5% glutaraldehyde at 4 °C overnight. The samples were sequentially exposed to a series of ethanol solutions (30, 50, 75, 90, 95, and 100% v/v) for 10 min, followed by vacuum freeze-drying overnight. SEM was then used to observe the surfaces of the samples after spraying gold.

#### Antibacterial longevity

To evaluate the longevity of the antibacterial activity, the samples were incubated in sterile modified Gamble’s solution [27] at 37 °C for 1 day and 21 days. The modified Gamble’s solution was refreshed every 5 days. At the appointed time points, the incubated samples were removed and immersed for 10 s in fresh modified Gamble’s solution, and an aliquot (100 μL) of the bacterial suspension containing 1 × 10^8^ CFU mL^−1^ of bacteria was dropped onto the sample surface and cultured for 18 h at 37 °C. Finally, the antibacterial activity was evaluated using the bacterial counting method described above.

### Cell adhesion test and platelet adhesion test

All materials were sterilized with ethylene oxide prior to use. Human normal lung epithelial cells (BEAS-2B cells) were seeded on the experimental material and the control material at a density of 2 × 10^5^ cells per well. After co-cultivation for 1, 4, and 7 days, the cells were gently washed with PBS three times and then stained with Live/Dead staining agent for 15 min in the dark. A CLSM was then employed to capture the cell adhesion images. ImageJ software (ImageJ, U.S. National Institutes of Health, Bethesda, MD) was used for cell counting and analysis.

Platelet-rich plasma (PRP) was separated from citrated whole blood via centrifugation at 1500 rpm for 5 min. A total of 500 μL of PRP was dropped on the surface of the samples and then incubated at 37 °C for 1 h. After this time, the attached platelets were lysed with 0.5% (v/v) Triton X-100 (2 mL) and quantified by testing the lactic dehydrogenase (LDH) absorbance at 490 nm, using an aliquot (500 μL) of PRP as a reference.

### Protein adhesion test

The PVC, PAAm-Gelatin and CS-AgNps@PAAm-Gelatin were brushed on the SiO_2_ chip prior to QCM. After allowing the baseline to stabilize under air, a modified Gamble’s solution was introduced. After stabilization of the modified Gamble’s solution baseline was stable, a modified Gamble’s solution containing the desired protein (i.e., BSA, HSA, or Cytochrome C) at a concentration of 1 mg mL^−1^ was introduced to observe the adsorption quality of the protein on the surfaces of the different coatings. Finally, the loosely bound protein was rinsed and removed using modified Gamble’s solution. Proteins with different isoelectric points (PI) were employed for the purpose of test, including BSA (PI = 4.8), HSA (PI = 4.9), and Cytochrome C (PI = 10.7), to study the anti-adsorption abilities of different proteins.

### Biocompatibility

The mouse embryonic fibroblast cell line (NIH 3T3 cells, ATCC) was used to assess the cytotoxicity, and material extracts were prepared after sterilizing the experimental materials with ethylene oxide. The experiment was divided into four groups: Blank, PVC (negative control), CS-AgNPs@PAAm-Gelatin, and phenol (positive control). The CS-AgNPs@PAAm-Gelatin coated PVC endotracheal tube was prepared into sterile small pieces with the size of 1 × 1 cm^2^ and the thickness of 2 mm, which were prepared with sterile cell culture medium according to the ratio of 1.25 cm^2^ mL^−1^, and stirred and extracted at 37 ℃ for 24 h. The extract of the negative control group was prepared by commercial PVC endotracheal tube according to the above process. The extract of the positive control group was prepared into 6.4 g mL^−1^phenol solution by dissolving phenol in the culture medium. The cells to be measured were arranged in a content of 5 × 10^3^ cells/well, and three multiple holes were set for each group. The cells were then cultured in a 5% CO_2_ incubator at 37 °C for 24 h. After the cells were completely attached to the well, the original medium was discarded, and the extract medium was added. After incubation for 1, 4 and 7 days, 10% CCK-8 medium (100 μL) was added to each well and incubation was carried out for a further 2 h. Then the absorbance value (A) at 450 nm was then measured with an Enzyme-linked detector, and the cell survival rate (%) was determined as follows:

$${\text{Cell survival rate }}\left( \% \right) = \frac{{\text{A value of the experimental group}}}{{\text{A value of the blank group}}} \times 100\%$$

To further study the biocompatibility of our system, the material extract was co-cultured with human normal lung epithelial cells (BEAS-2B cells) for 48 h prior to nuclear staining and Live/Dead staining. Finally, the cell morphologies were examined under an inverted fluorescence microscope.

### The artificial airway-lung mechanical ventilation model

A fixture was designed to simultaneously evaluate the antifouling properties of the two ETTs, as shown in Fig. 7a. In all experiments, the ETT angle was set at 30°, which was consistent with the current clinical situation [21]. Two ETTs were connected to the ventilator through an air separator and a breathing circuit, and placed in a liquid storage tank filled with artificial mucus. The test lung connected to the air tank returned air to the ventilator, simulating the breathing of a patient. The ventilator was set to the pressure control mode with a breathing rate of approximately 10 breaths/min. The maximum inspiratory pressure was 10 cm H_2_O, the positive end expiratory pressure (PEEP) was 4 cm H_2_O, and the inspiratory time was 0.6 s. The composition of the artificial mucus was as follows: 4% w/v mucin from porcine stomach, Type II (Sigma-Aldrich, St. Louis, MO), 2% w/v lecithin (Alfa Aesar, War Hill, MA, USA), 40 mM potassium hydroxide (dibasic), 20 mM potassium hydroxide (monobasic), 50 mM ammonium sulfate, 1 mM magnesium sulfate (Sigma-Aldrich, St. Louis, MO), 50 U mL^−1^ each of penicillin/streptomycin, and 1 μg mL^−1^ Fungizone^®^ antimycotic (Life Technologies, Carlsbad, CA) [28, 29]. All instruments were heated to 37 °C for the purpose these experiments. After continuous ventilation for 48 and 72 h, the adhesion properties of the different ETTs were observed.

### Porcine mechanical ventilation model with oropharyngeal *P. aeruginosa* challenge

Eight Large White pigs (weight range, 40–45 kg) were purchased from the Shanghai Jiagan Experimental Animal Raising Farm. All experimental protocols were approved by the Animal Experimental Ethical Inspection of Shanghai Jiagan Biotechnology Co. Ltd. (JGLL2021110). The pigs were premedicated with an intramuscular dose of 0.2 mg kg^−1^ of xylazine hydrochloride and induced with an intravenous loading dose of 8 mg kg^−1^ sodium thiopental. Pigs were then orotracheally intubated with a 6.0-mm I.D.PVC-ETT and CS-AgNps@PAAm-Gelatin-ETT respectively, and mechanically ventilated for 48 h. Continuous inhalation of 2% isoflurane and infusion of propofol (2–4 mg kg^−1^ h^−1^) was carried out, and each animal was challenged after 1 and 4 h of tracheal intubation. An aliquot (5 mL) of ~ 10^8^ CFU mL^−1^ of a log-phase culture of *P. aeruginosa* or *S. aureus* was slowly instilled into the oropharynges of the animals. Within 10 min of adding the bacterial challenge, a PEEP of 5 cm H_2_O was applied to avoid rapid aspiration of the pathogens. During the ventilation process, the airway pressure of the experimental pigs did not exceed 15 cm H_2_O. After 48 h of tracheal intubation (40 h after the bacterial challenge), the animals were euthanized. The animals were positioned supine, exposed under strictly sterile conditions, and the lungs were excised and placed on sterile drapes. Samples from each of the five lobes and the tracheal endothelium were used for histological and microbiological assessments. In addition, the internal mucus adhesions of the different ETTs were observed.

### Statistical analysis

All experiments were conducted in at least three independent replicates. Prism 8.2 software (GraphPad, La Jolla, CA, USA) was used for statistical analyses. The data were expressed as means ± standard deviation (SD), and statistical analysis between the different groups was performed using one-way analysis of variance (ANOVA). The statistical significance levels are represented by ****p < 0.0001, ***p < 0.001, **p < 0.01, and *p < 0.05.

## Results and discussion

### Characterization of the prepared CS-AgNps@PAAm-Gelatin coating

**Fig. 1:**
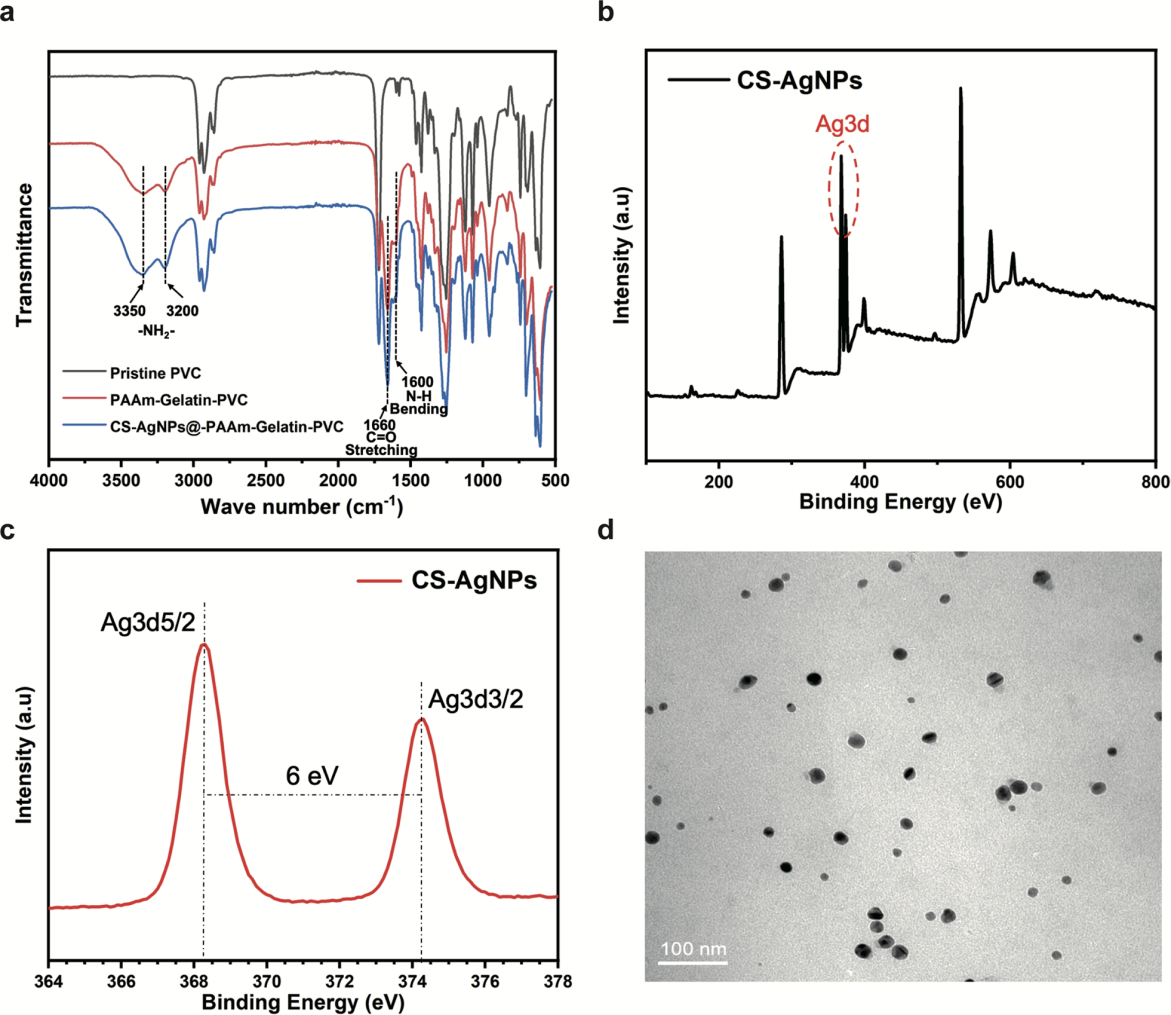
Characterization of the CS-AgNPs@PAAm-Gelatin nanocomposite coating. **a** Fourier transform infrared spectra of CS-AgNPs@PAAm-Gelatin. **b** XPS wide scanning and fine scanning (**c**) of the CS-AgNPs. **d** TEM images of CS-AgNPs (scale bar = 100 nm)

The acrylamide was subjected to in situ polymerization in the gelatin network to form a PAAm molecular chain gel interpenetrating polymer network (IPN) architecture, in which the CS-AgNPs were uniformly dispersed. To confirm the structure of the composite, the coated PVC ducts, individual coatings, and individual antibacterial agents were characterized. More specifically, the FTIR spectra of the PAAm-Gelatin-PVC and CS-AgNPs@PAAm-Gelatin-PVC samples showed apparent absorption peaks at 3200–3350 cm^−1^, which indicated the presence of amino groups. The absorption peak at 1660 cm^−1^ was ascribed to the carbonyl (C=O) groups, while the peak at 1600 cm^−1^ was assigned to the imino (N–H)-anchored polyacrylamide chains. These FTIR spectral observations suggested that all expected functional groups were present in the polymeric gelatin networks, and that the AgNPs had no significant influence on the structure (Fig. 1a). The FTIR spectra of the PAAM-PVC coating showed characteristic peaks similar to Fig. 1a, that is, the absorption peaks at 3200–3350 cm^−1^ still existed, which conformed that the interaction between PAAm and PVC could be strong covalent anchorage and was in independent of gelatin (Additional file 1: Figure S1).

The type and crystal form of the AgNPs were determined by XRD. As shown in Additional file 1: Figure S2, a widened diffraction peak appeared at 38.1, which represented the existence of silver (111) crystal plane in face centered cubic crystal form [30]. In cooperation with the lattice fringe analysis of CS-AgNPs collected by high-resolution transmission electron microscope (HRTEM), the lattice spacing was 0.235 nm (Additional file 1: Figure S3), which was completely matched with the silver (111) crystal plan. The peak intensity and width of the diffraction peak implied that the AgNPs were fixed to CS or covered by CS, and the nanoparticle size was very small. After understanding the crystal structure of silver nanomaterials, we characterized the particle size, morphology and optical properties of the materials by TEM and UV–Vis. The average diameter of nanoparticles was 16.71 ± 4.84 nm and the particles were homogeneous and uniform (Fig. 1d, Additional file 1: Figure S4). The UV–Vis spectrophotometric changes of antibacterial solution containing different concentrations showed that the absorption peak only gradually increases with the increase of proportion of nano silver, indicating the effective introduction of nano silver (Additional file 1: Figure S5). The single absorption peak of CS-AgNPs appears near 420 nm, which was consistent with the characteristic absorption peak of silver nanoparticles [31]. CS-AgNPs were further characterized by XPS. The silver single substance peak was located by wide scanning (Fig. 1b), and then the fine scanning showed that there were binding peaks between 3d5/2 and 3d3/2 orbits, and the peak energy was approximately 6 eV, indicating that silver existed in the form of single substance (0 valence) (Fig. 1c).

**Fig. 2:**
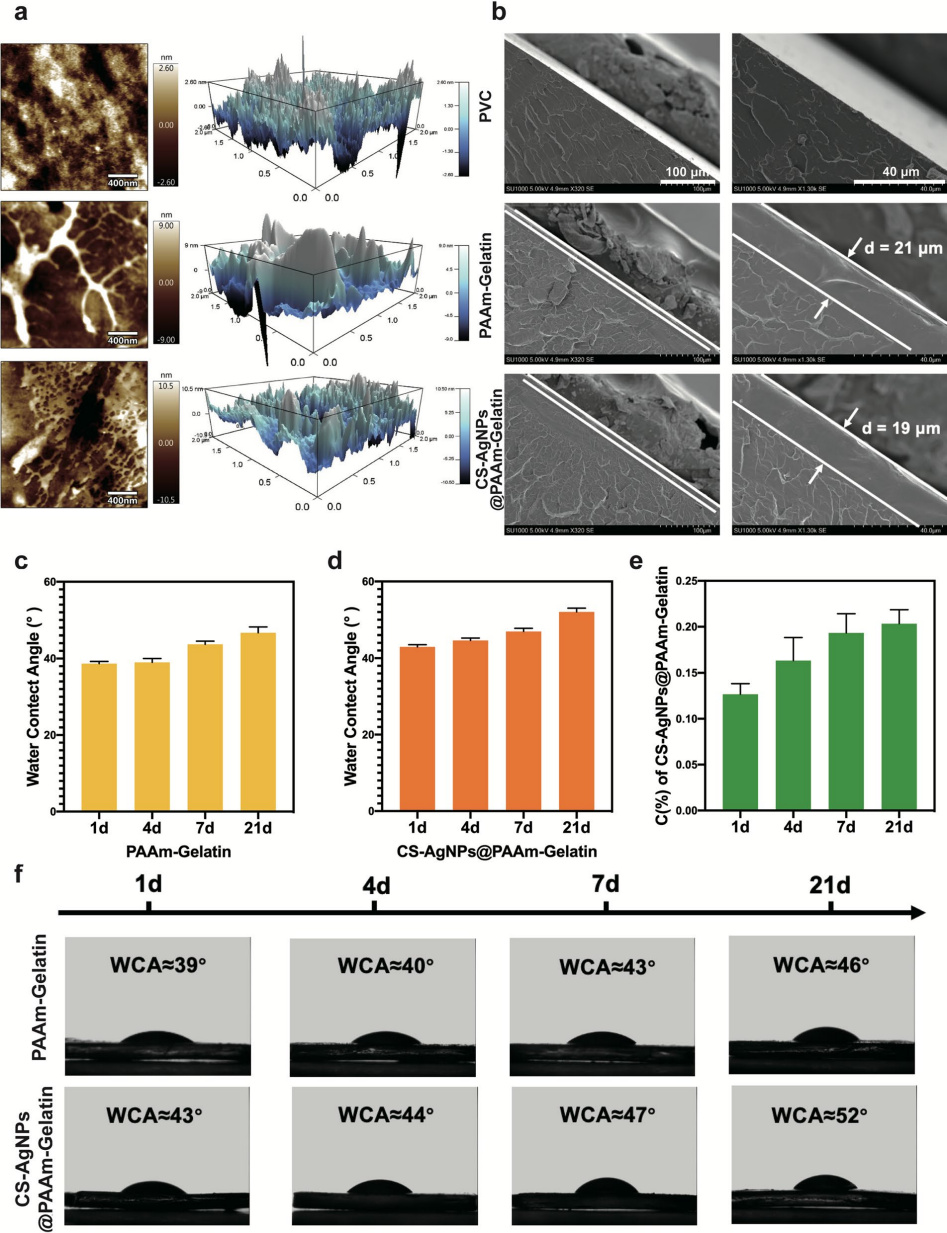
Surface morphology and water contact angles. **a** AFM images of PVC, PAAm-Gelatin, and CS-AgNps@PAAm-Gelatin samples. **b** SEM images of PVC, PAAm-Gelatin, and CS-AgNps@PAAm-Gelatin samples. Changes of water contact angles of PAAm-Gelatin (**c**) and CS-AgNps@PAAm-Gelatin (**d**) after immersion in modified Gamble’s solution at 37 ℃ after 1, 4, 7, 21 days. **e** Carbon (C) contents of CS-AgNps@PAAm-Gelatin after immersion in modified Gamble’s solution at 37 ℃ for 1, 4, 7, and 21 days. **f** Micrographs of water droplets on the PAAm-Gelatin and CS-AgNps@PAAm-Gelatin samples after 1, 4, 7, and 21 days, respectively

The synthesized CS-AgNPs@PAAm-Gelatin-PVC also exhibited clear peaks related to the amide groups, indicating that the interaction between PAAM and the PVC substrate was strong covalent anchoring rather than a weak deposition or absorption of the PAAm chain. Ag ions were fixed by ordered amino groups on CS surface and AgNPs were formed in situ under the action of reducing agent. In view of this special surface state, the material may release silver ions slowly and have lasting antibacterial properties. AFM and SEM were then used to examine the surface morphology of the NPs, wherein the AFM images intuitively displayed the change in surface roughness (Fig. 2a), and the coating thickness was calculated using material section electron microscope imaging. The thickness of the CS-AgNPs@PAAm-Gelatin coating was calculated to be 15–40 μm (Fig. 2b), which had little effect on the inside diameter of the commercial PVC-ETT. It is worth noting that no significant differences were observed in the appearances of the commercial PVC-ETT and CS-AgNPs@PAAm-Gelatin-ETT samples (Additional file 1: Figure S9). To test the stability of the coatings, the water contact angle (WCA) measurement and element analysis were carried out. The PVC, PAAm-Gelatin-PVC and CS-AgNPs@PAAm-Gelatin-PVC coating immersed in modified Gamble’s solution at 37 ℃ for 1, 4, 7, and 21 days. The PVC contact angles were approximately stable at 95° at all times (Additional file 1: Figure S6). These results demonstrate that the WCA of PAAm-Gelatin-PVC only increased by− 7° over 21 days (Fig. 2c), which suggested the surface hydrophilicity was stable. Similar results were obtained after the introduction of the antibacterial agent. For example, the WCA of CS-AgNPs@PAAm-Gelatin-PVC increased by − 9° over 21 days (Fig. 2d), further confirming the stability of the coating, and indicating that the antibacterial agent had no effect on the coating surface hydrophilicity (Fig. 2f). In addition, according to the elemental analysis of modified Gamble’s solution, the main element carbon in the CS-AgNPs@PAAm-Gelatin coating did not dissolve within 21 days, which further indicated the strong stability of the coating (Fig. 2e). Moreover, tensile tests were performed to characterize the mechanical properties of the materials, and the average tensile strains of 185 and 190% obtained for PVC-ETT and CS-AgNPs@PAAm-Gelatin-ETT indicated that the mechanical properties of the ETT were not affected by the CS-AgNPs@PAAm-Gelatin coatings (Additional file 1: Figure S7, 8).

### Antibacterial activity

The initial exposure of patients to pathogenic microorganisms that cause VAP usually occurs through the bacterial colonization of medical devices. The most common pathogens associated with VAP include *S. aureus* and *P. aeruginosa*. Once a pathogen is established, it can initiate a disease state in a patient, especially in critically ill patients. When organisms possess the phenotypic ability to form biofilms, the infection intensifies. In addition, the increase in cell density enhances the effect of quorum sensing, causing genetic mutations that reduce the metabolic activity; in other words, a combative environment increases the phenotypic variation between bacteria, thereby providing favorable defense and progress, ultimately leading to antibiotic resistance [32, 33]. Biofilm formation on the surfaces of medical devices is therefore a key feature of disease progression, and so the antiadhesion and antibacterial functionalization of PVC-ETTs with hydrophilic polymers and stable broad-spectrum antibacterial agents is particularly important in the prevention of biofilm formation and bacterial infection [34–37]. It is therefore also critical to suppress bacterial adhesion and multiplication during the initial stage.

As mentioned above, Ag ions were immobilized by amino groups on the surface of the CS, and AgNPs were formed in situ under the action of a reducing agent. The sustained release of the antibacterial agent (i.e., Ag^+^) is an important characteristic of Ag^+^-loaded coatings. As shown in Additional file 1: Figure S10, the total quantity of loaded Ag^+^ in the CS-AgNPs@PAAm-Gelatin coating was 75 μg cm^−2^, and a relatively low dosage release was achieved within 110 h. Moreover, the amount of Ag^+^ released from the coating was 6.975 μg cm^−2^ (9.3% of the total content), which also supported the longevity of the antibacterial activity.

**Fig. 3:**
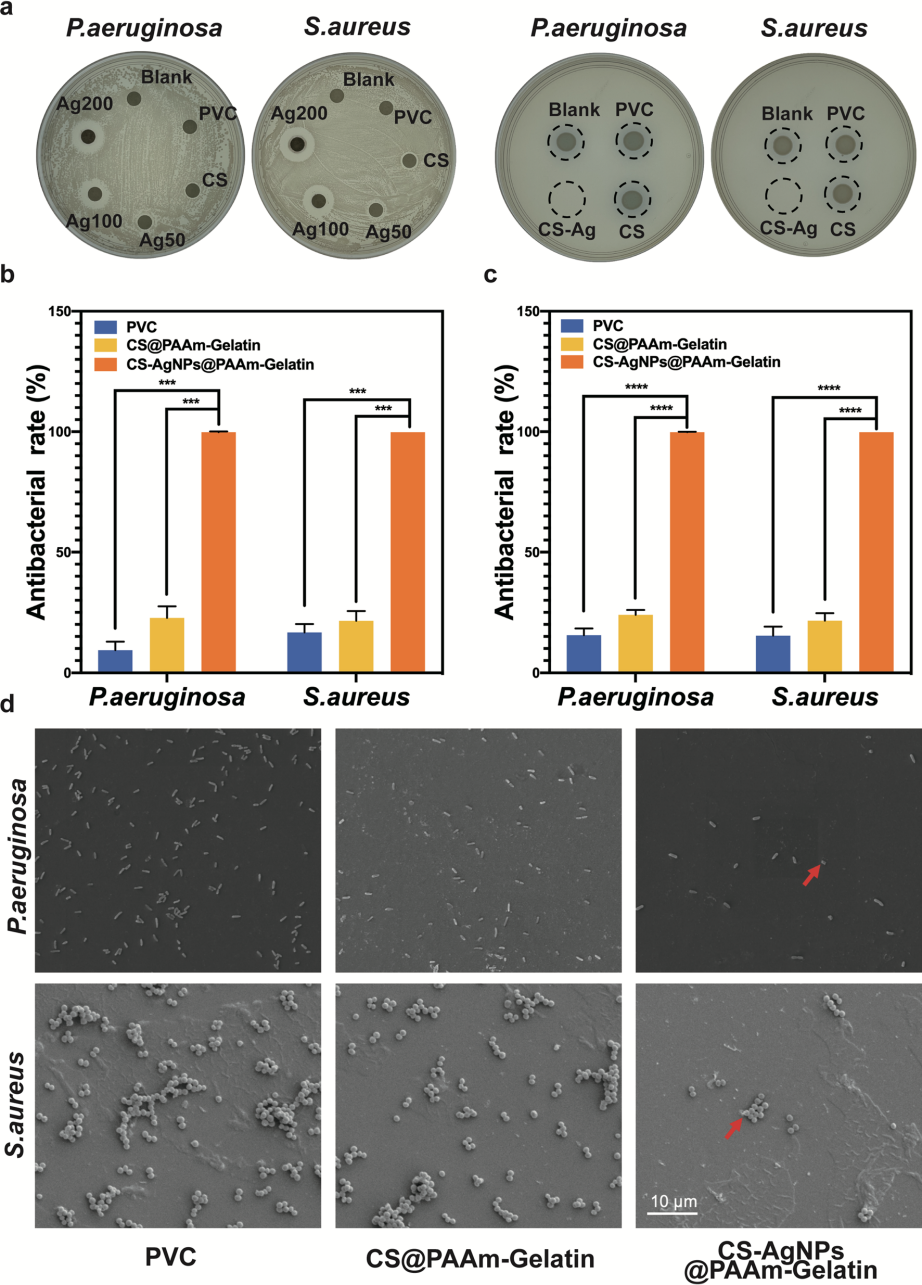
In vitro antibacterial performance. **a** Survival of bacterial colonies and inhibition zones in the inherent antibacterial test. The quantitative antibacterial ratio of various samples for *P. aeruginosa* and *S. aureus* after 1 day (**b**) and 21 days incubation in modified Gamble’s solution (**c**). **d** Morphology changes of *P. aeruginosa* and *S. aureus*. The red arrows indicate pathogens with morphological changes. The data are expressed as means ± SD (n = 3). (***p < 0.001, ****p < 0.0001)

The antibacterial capability of the CS-AgNPs@PAAm-Gelatin-ETT system against *P. aeruginosa* and *S. aureus* was then assessed, with commercial PVC-ETTs being used as controls. Initially, we performed an agar diffusion assay to determine the optimal antibacterial agent concentration. The different concentrations of antibacterial agent were diluted to CS-AgNPs content of 50, 100, 200 ppm, and 6 mm filter papers stained with diluted antibacterial agent were labeled as Ag50, Ag100, and Ag200. The diameter of the zone of inhibition in *P. aeruginosa* and *S. aureus* cultured with Ag200 were 14.5 and 16.8 mm, respectively (Fig. 3a). Thus, we fabricated CS-AgNPs@PAAm-Gelatin-PVC with an AgNP content of 200 ppm to carry out the following experiments, wherein commercial PVC and CS@PAAm-Gelatin-PVC were selected as the control. As a result, almost no bacterial colonies were observed after co-culture with the CS-AgNPs@PAAm-Gelatin-PVC system (Fig. 3a), thereby indicating the excellent bactericidal performance of our coating (Fig. 3b). Finally, to evaluate the longevity of the antibacterial activity, all samples were incubated in modified Gamble’s solution at 37 °C for 21 days, and it was found that after this time, CS-AgNPs@PAAm-Gelatin-PVC continued to present an antibacterial ratio of 99% for both bacteria while CS@PAAm-Gelatin-PVC only presented an antibacterial ratio of 25% (Fig. 3c). Additionally, morphology changes were observed both in *P. aeruginosa* and *S. aureus* on the surface of different samples. Compared with commercial PVC and CS@PAAm-Gelatin-PVC, the two bacteria on the surface of CS-AgNps@PAAm-Gelatin-PVC exhibited damaged cellular morphology (Fig. 3d).

### Cell adhesion test

**Fig. 4:**
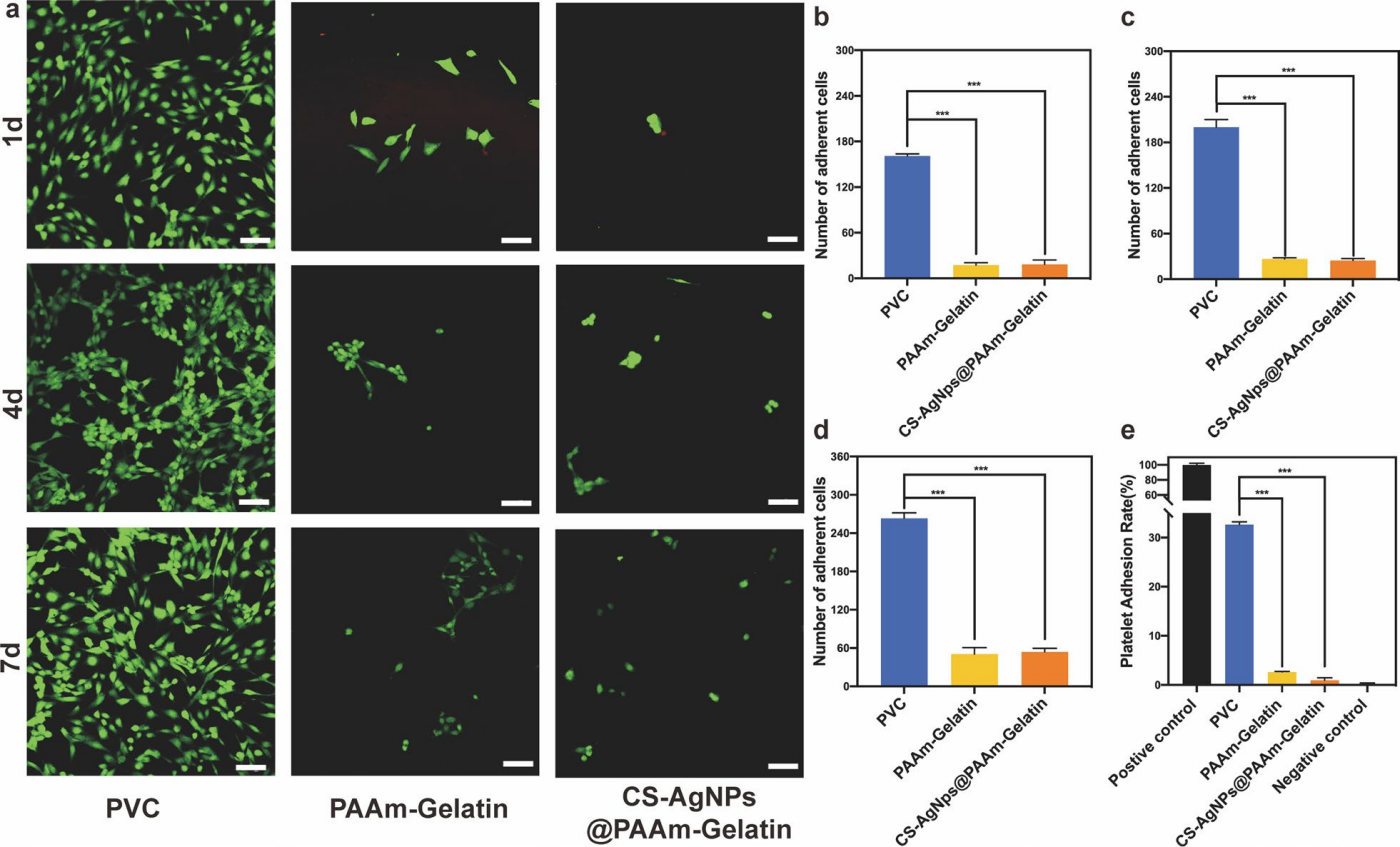
Anti-adhesion assays. **a** Live/Dead staining of adherent cells (scale bar = 100 μm). **b**–**d** Adhesion amounts following co-cultivation for 1, 4, and 7 days, respectively. **e** Platelet adherence on the various samples. All data are expressed as means ± SD (n = 3) (***p < 0.001)

The main components of airway secretions are shed epithelial cells, proteins, bacteria, and blood. The anti-adhesion properties of the coating are therefore particularly important as secretions often adhere to ETTs, resulting in inadequate ventilation or biofilm formation. It is well known that hydrophilic surfaces are not conducive to the adhesion of microorganisms such as cells [38–40], while the positive charge on the surface of the material is conducive to the adsorption of the negatively charged cell membrane. It was found that coating with CS-AgNPs@PAAm-Gelatin led to an increase in the surface potential from − 35 mV to almost 0, without the formation of a positive potential (Additional file 1: Figure S11). Subsequently, BEAS-2B cells were seeded onto the CS-AgNPs@PAAm-Gelatin, PAAm-Gelatin, and PVC surfaces. After co-cultivation for 1, 4, and 7 days, the adhesion amount of PVC was found to far exceed those observed for the other two groups (*p* < 0.001) (Fig. 4a), indicating that the PAAm-Gelatin cross-linked network system exhibits an excellent anti-adhesion performance even after the introduction of the antibacterial agent (Figs. 4b–d).

Platelets are the most important adhesion components in blood cells, and in this context, it should be noted that the sugar-based coating on the surface can adsorb plasma proteins and coagulation factors. When blood vessels are damaged or ruptured, the surface viscosity of the platelets increases and aggregation takes place to form clusters [41]. Thus, the effects of PAAm-Gelatin and CS-AgNPs@PAAm-Gelatin on platelet attachment were also evaluated, with commercial PVC being used as the control group. It was found that the platelet adhesion rate of PVC was 32.67 ± 0.77%, while that of PAAm-Gelatin was 2.63 ± 0.17%. In contrast, the platelet adhesion rate of CS-AgNPs@PAAm-Gelatin was ~ 0.95 ± 0.60% (*p* < 0.001). These results therefore confirm that the CS-AgNPs@PAAm-Gelatin coating exhibited a superior performance in terms of reducing platelet attachment and maintaining the anti-adhesion capacities toward the different cells (Fig. 4e).

### Protein adhesion test

**Fig. 5:**
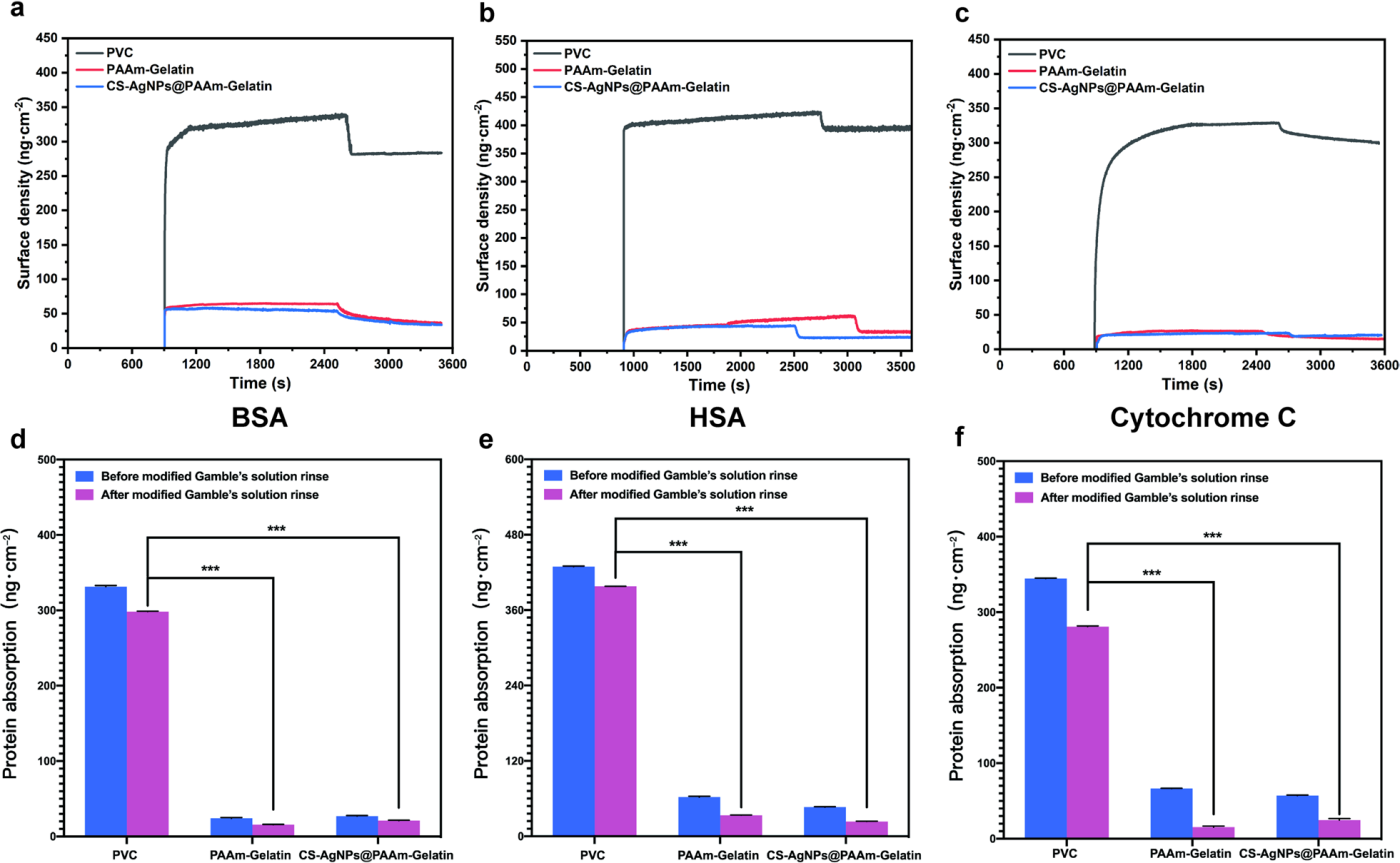
Protein adhesion test. **a**–**c** Representative time tracks of QCM-D crystal sensors with different coatings in BSA, HSA and Cytochrome C protein solutions, respectively. **d**–**f** Different protein adsorption of BSA, HSA and Cytochrome C onto the PVC, PAAm-Gelatin, CS-AgNps@PAAm-Gelatin surfaces before and after modified Gamble’s solution rinse. The data were expressed as means ± SD (n = 3). (***p < 0.001)

QCM-D was used to evaluate the dynamic adsorption of BSA (PI = 4.8), has (PI = 4.9), and Cytochrome C (PI = 10.7) on the different coatings. To verify the antifouling effect of PAAm and the gelatin interpenetrating network structure, PVC, PAAm-Gelatin, and CS-AgNPs@PAAm-Gelatin were tested. Using the Voigt viscoelastic model, the specific protein adsorption quality was simulated and calculated. Following introduction of the modified Gamble’s solution of BSA, the adsorption amounts of all surface proteins gradually increased to their maximum values, indicating that BSA was gradually adsorbed on the surfaces. The mass of BSA adsorbed by PVC was ~ 333 ng cm^−2^, while the corresponding values for by PAAm-Gelatin and CS-AgNPs@PAAm-Gelatin were 25 and 28 ng cm^−2^, respectively. After rinsing with modified Gamble’s solution to remove any loosely bound BSA, the remaining BSA adsorbed by PVC was determined to be 299 ng cm^−2^, while PAAm-Gelatin and CS-AgNPs@PAAm-Gelatin gave values of 15 and 21 ng cm^−2^, respectively (Fig. 5a). Thus, compared with PVC, PAAm-Gelatin decreased protein adsorption by 95%, while CS-AgNPs@PAAm-Gelatin decreased protein adsorption by 93%, thereby indicating its considerable antifouling ability. Also, HSA and Cytochrome C were tested and the results were consistent with BSA, which showed anti-protein adsorption rate from 90 to 94% after rinsing with modified Gamble’s solution. (Fig. 5b, c, e, f).

### Cytotoxicity assessment in vitro

**Fig. 6:**
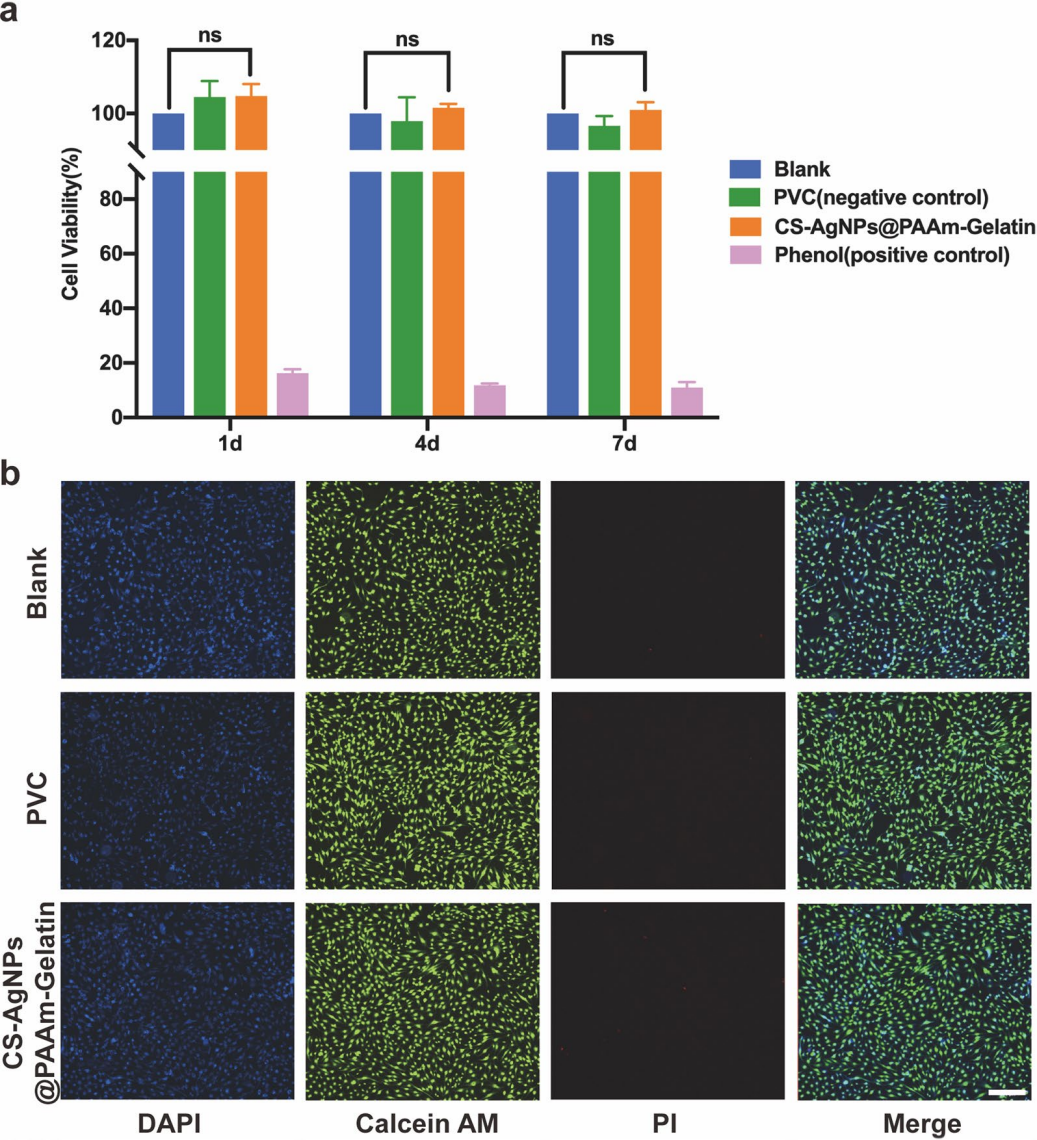
Cytotoxicity of CS-AgNps@PAAm-Gelatin. **a** Quantitative CCK-8 assay after incubation for 1, 4, and 7 days, respectively. **b** Live/Dead staining after the BEAS-2B cells were seeded onto CS-AgNPs@PAAm-Gelatin (scale bar = 100 μm). All data are expressed as means ± SD (n = 3) (ns represented not significant)

The CCK-8 kit assay and Live/Dead staining assay were conducted to evaluate the in vitro cytotoxicity of CS-AgNPs@PAAm-Gelatin. In the CCK-8 assay, the viability of NIH L929 cells was in the range of 101.01 ± 2.07–104.77 ± 3.27% after incubation for 1, 4, and 7 days (Fig. 6a), indicating the negligible cytotoxicity of CS-AgNPs@PAAm-Gelatin, and suggesting that it could be safely used for coating ETTs. Besides, BEAS-2B cells were co-cultured with the extract of CS-AgNPs@PAAm-Gelatin for 48 h, and then mixed with Calcein AM and PI dye solutions. As shown in Fig. 6b, the cells proliferated and were morphologically intact, with no evidence of cell death being observed. Moreover, pigs with CS-AgNPs@PAAm-Gelatin-ETT intubations in mechanical ventilation model with oropharyngeal *P. aeruginosa* and *S. aureus* challenge showed no significant changes in their tracheal endothelia compared to pigs with PVC-ETT intubations after mechanical ventilation for 48 h (Additional file 1: Figures S12, 13).

### Artificial airway-lung mechanical ventilation model

**Fig. 7:**
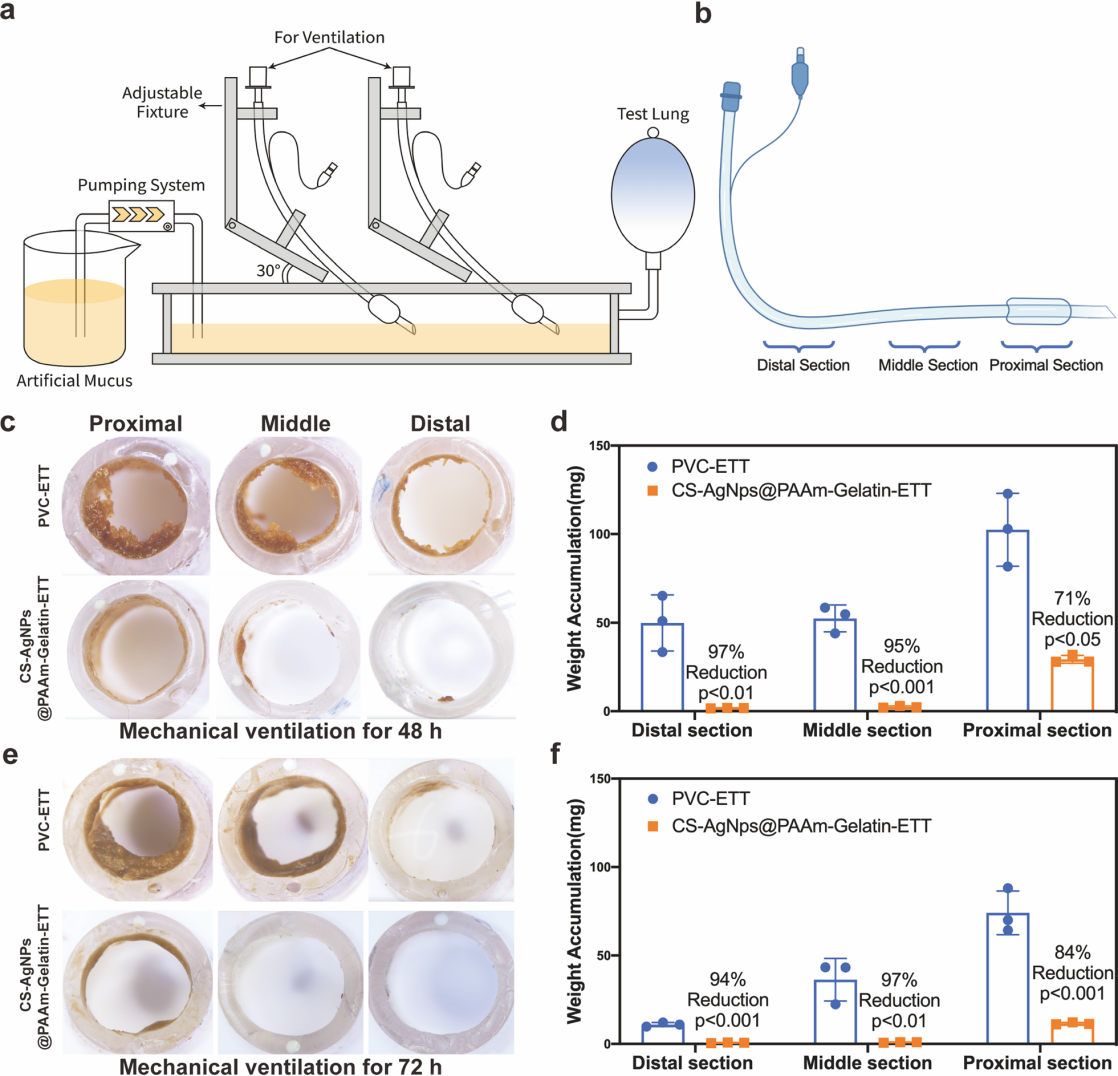
Artificial airway-lung mechanical ventilation model. **a** Plot for the artificial airway-lung mechanical ventilation model showing the artificial mucus weights in the ETTs grouped by section along the tube length. **b** The proximal section was 1–4 cm from the top of the ETT, the middle section was 12–15 cm from the top of the ETT, and the distal section was 22–25 cm from the top of the ETT. **c**, **d** Different sections of PVC-ETT and CS-AgNPs@PAAm-Gelatin-ETT artificial mucus accumulation after mechanical ventilation for 48 h. **e**, **f** Different sections of PVC-ETT and CS-AgNPs@PAAm-Gelatin-ETT artificial mucus accumulation after mechanical ventilation for 72 h. All data are expressed as means ± SD (n = 3)

As shown in Fig. 7a, the artificial airway-lung mechanical ventilation model was used to evaluate the antifouling properties of the CS-AgNPs@PAAm-Gelatin-ETTs, while the PVC-ETTs were used as the control group. After mechanical ventilation for 48 and 72 h, comparison of the in vitro mucus weights for the CS-AgNPs@PAAm-Gelatin-ETTs and PVC-ETTs systems yielded significant reductions in the various measured sections. The yellow areas inside the luminal portion of each ETT section accumulated artificial mucus. The proximal section was 1–4 cm from the top of the ETT, the middle section was 12–15 cm from the top of the ETT, and the distal section was 22–25 cm from the top of the ETT (Fig. 7b). After mechanical ventilation for 48 and 72 h, the adhesion of artificial mucus to the ETTs was observed, and it was found that the CS-AgNPs@PAAm-Gelatin-ETTs showed significantly reduced decreases in the cross-sectional areas of all three sections along the tubes compared to those observed for the PVC-ETTs. Furthermore, quantitative analysis of the weight accumulation after mechanical ventilation for 48 h demonstrated decreased lumen occlusions of 97% (*p* < 0.01) in the distal sections, 95% (*p* < 0.001) in the middle sections, and 71% (*p* < 0.05) in the proximal sections (Figs. 7c, d). In addition, after mechanical ventilation for 72 h, the CS-AgNPs@PAAm-Gelatin-ETTs continued to exhibit excellent anti-adhesion properties, with a decreased lumen occlusion of 94% (*p* < 0.001) in the distal sections, 97% (*p* < 0.01) in the middle sections, and 84% (*p* < 0.001) in the proximal sections (Figs. 7e, f), further indicating the good stability of the CS-AgNPs@PAAm-Gelatin coating.

### A porcine mechanical ventilation model with oropharyngeal *P. aeruginosa* and *S. aureus* challenge

In this study, an animal VAP model was established to evaluate the antibacterial and antifouling functions of CS-AgNps@PAAm-Gelatin-ETT in vivo [42], the commercial PVC-ETT was used as a control. The main pathogenic mechanism of VAP is the inhalation of oropharyngeal secretions colonized by *P. aeruginosa* and *S. aureus* through the lungs. The VAP model employed herein has several advantages, including a good repeatability and easy induction. It is therefore capable of reflecting human etiology and possessing a consistent infection time.

**Fig. 8:**
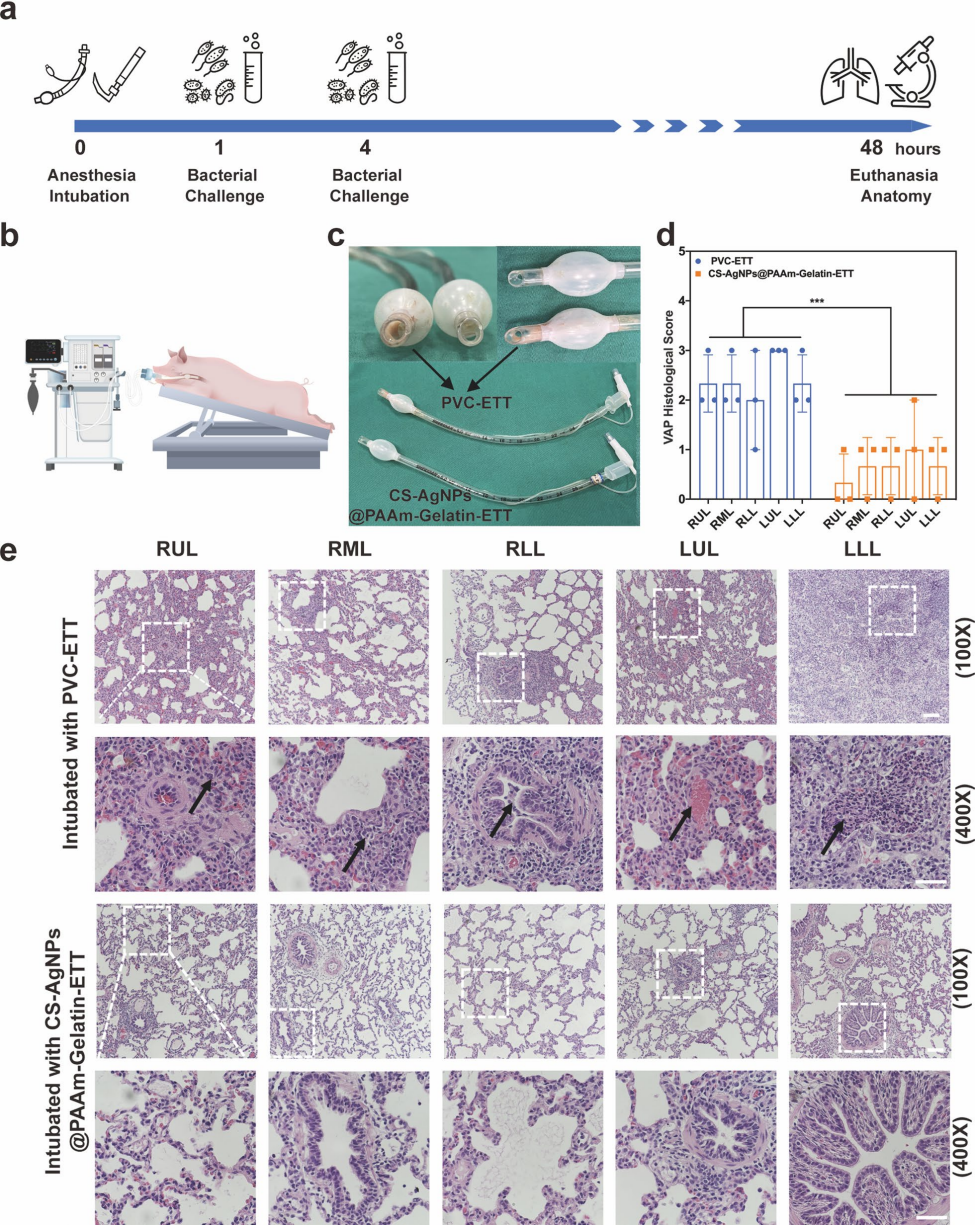
Porcine mechanical ventilation model with the oropharyngeal *P. aeruginosa* challenge. **a** Timeline of the porcine mechanical ventilation model. **b** Animal position during 48 h of mechanical ventilation. The pigs were placed in the prone position, and the surgical bed was oriented at − 30° to achieve an orientation of the respiratory system corresponding to the semi-recumbent position in humans. **c** Mucus adhesion to the ETTs after 48 h of mechanical ventilation. The PVC-ETT was clearly blocked by purulent secretions, while the CS-AgNPs@PAAm-Gelatin-ETT was clean and unobstructed. **d** VAP histological score of pigs intubated with PVC-ETTs and CS-AgNPs@PAAm-Gelatin-ETTs: 0, no injury; 1, purulent mucous plugging; 2, bronchiolitis; 3, pneumonia; 4, confluent pneumonia; and 5, abscessed pneumonia (***p < 0.001). **e** All pulmonary sections were stained with hematoxylin and eosin (H&E). Right upper lobe (RUL), right middle lobe (RML), right lower lobe (RLL), left upper lobe (LUL), and left lower lobe (LLL). H&E staining of lungs from a pig intubated with PVC-ETT. The arrows (RUL, RML, LLL) represented that extensive polymorphonuclear infiltration was found within the alveolar spaces. A purulent mucus plug was found and arrowed within the bronchiole lumen with surrounding inflammatory infiltration (RLL). Abnormal accumulations of red blood cells were observed and arrowed in the alveoli (LUL). H&E staining of lungs from a pig intubated with CS-AgNPs@PAAm-Gelatin-ETT. All five lung lobes possessed intact alveolar walls with no inflammatory infiltration (RUL). No mucous blockage was observed in the bronchioles (RML, RLL) or the terminal bronchioles (LUL, LLL) (100 × scale bar = 100 μm, 400 × scale bar = 50 μm)

Timeline of the porcine mechanical ventilation model and animal position during 48 h of mechanical ventilation were presented (Fig. 8a, b). As shown in Fig. 8c, significant differences were observed in mucus adhesion between the different ETTs after 48 h of mechanical ventilation. More specifically, the inner surface of the PVC-ETT was partly blocked by purulent secretions, which were mainly found in the proximal section. In contrast, the CS-AgNPs@PAAm-Gelatin-ETT was clean and unobstructed. In addition, pigs with PVC-ETT intubation experienced a gradual increase in body temperature and an increased thickening of their respiratory secretions within 20 h after the first bacterial attack, which indicated the possibility of inflammation. However, pigs with CS-AgNPs@PAAm-Gelatin-ETT intubations showed no significant changes in their body temperature or respiratory secretions after bacterial attack (Additional file 1: Figure S14). Furthermore, the lungs of pigs with PVC-ETT intubations showed extensive neutrophil infiltration with diffuse alveolar damage and hyaline membranes, along with mucus blockage in the bronchioles, which indicated the occurrence of VAP. These characteristic pathological changes were indicated by arrows. In contrast, in the group of pigs with CS-AgNPs@PAAm-Gelatin-ETT intubations, the lung tissue structure was intact, with little damage being observed overall. Moreover, the alveolar wall was normal without any inflammatory infiltration, and little mucus blockage was observed in the bronchioles (Fig. 8e). Additionally, the VAP histological scores of the different groups were significantly different, which again demonstrated that the lungs of pigs with PVC-ETT intubations were more seriously damaged than those with CS-AgNPs@PAAm-Gelatin-ETT intubations (Fig. 8d). Porcine mechanical ventilation model with the oropharyngeal *S. aureus* challenge had similar results to *P. aeruginosa* challenge (Additional file 1: Figure S15). Gram staining was also performed on the tissue sections, but no relevant staining was found. We used *P. aeruginosa* and *S. aureus* to induce VAP, in which the lung tissue was extensively infiltrated with inflammatory cells accompanied by the body’s own defense mechanisms. For example, the goblet cells at all levels of the trachea, Clara cells, and macrophages in lung stroma could secrete mucin and phagocytose pathogens, which may result in the pathogen being enveloped and unable to be stained. The results of the porcine mechanical ventilation model with oropharyngeal *P. aeruginosa* and *S. aureus* challenge therefore implied that CS-AgNPs@PAAm-Gelatin-ETTs not only exhibited anti-adhesion properties, but that they also exhibited an excellent antibacterial performance, which could effectively prevent VAP or reduce its severity.

## Conclusion

Surface modification is a useful strategy for enhancing the biological properties of endotracheal tubes (ETTs). Thus, we herein reported the preparation of a CS-AgNPs@PAAm-Gelatin nanocomposite for application as a coating on the internal and external surfaces of ETTs. The interpenetrating structure of the PAAm molecular chain gel network in coatings enhanced the hydrophilicity of the substrate, and prevented the adhesion of proteins, cells, bacteria, and other microorganisms. Both in vitro and in vivo results showed that the CS-AgNPs@PAAm-Gelatin coatings exhibited excellent antibacterial properties to prevent ventilator-associated pneumonia, as well as showing a good biocompatibility and stability. Additionally, the synthetic process employed to obtain the coating material is environmentally friendly, and the process of anchoring the coating to polyvinyl chloride is simple and reliable, and shows great potential for future clinical application.

## Supplementary Information

**Additional file 1: Figure S1.** Fourier transform infrared spectra of PAAm-PVC. **Figure S2.** XRD spectra of the CS-AgNps@PAAm-Gelatin. **Figure S3.** Spacing of Ag (111) lattice fringe (d = 0.235 nm). **Figure S4.** The size distribution of CS-AgNPs. **Figure S5.** UV-visible spectra of silver-chitosan nanocomposite colloids prepared using different AgNO_3_ concentrations. **Figure** **S6.** Water contact angles of PVC. **Figure S7.** Stress-strain curves for the PVC-ETT (black) and CS-AgNps@PAAm-Gelatin-ETT (red) specimens. **Figure S8.** Standard process for the tensile test. **Figure S9. **General views of the commercial PVC-ETT and CS-AgNps@PAAm-Gelatin-ETT specimens. Observations of CS-AgNps@PAAm-Gelatin-ETT showed that the colorless coating made it indistinguishable from PVC-ETT, and no streaks or spots were observed on any surface. It was important that the cuff of the CS-AgNps@PAAm-Gelatin-ETT was not affected by the dip coating process, and could be inflated and deflated repeatedly to ensure its airtightness. **Figure S10.** Released amounts of Ag^+^ from CS-AgNPs@PAAm-Gelatin after immersion in PBS for up to 110 h. **Figure S11.** Zeta potential of PVC, PAAm-Gelation, and CS-AgNPs@PAAm-Gelatin. **Figure S12. **Porcine mechanical ventilation model with the oropharyngeal *P. aeruginosa* challenge. The tracheal endothelium was stained with hematoxylin and eosin (H&E). No sections exhibited inflammatory infiltration. (Scale bar = 100 μm). **Figure S13. **Porcine mechanical ventilation model with the oropharyngeal S. aureus challenge. The tracheal endothelium was stained with hematoxylin and eosin (H&E). No sections exhibited inflammatory infiltration. (Scale bar = 100 μm). **Figure S14. **Body temperature of pigs intubated with PVC-ETT and CS-AgNPs@PAAm-Gelatin-ETT respectively. **Figure S15.** Porcine mechanical ventilation model with the oropharyngeal *S. aureus* challenge. **a** Mucus adhesion to the ETTs after 48 h of mechanical ventilation. The PVC-ETT was clearly blocked by purulent secretions, while the CS-AgNPs@PAAm-Gelatin-ETT was clean and unobstructed. **b** VAP histological score of pigs intubated with PVC-ETTs and CS-AgNPs@PAAm-Gelatin-ETTs: 0, no injury; 1, purulent mucous plugging; 2, bronchiolitis; 3, pneumonia; 4, confluent pneumonia; and 5, abscessed pneumonia (****p < 0.0001). **c** All pulmonary sections were stained with hematoxylin and eosin (H&E). Right upper lobe (RUL), right middle lobe (RML), right lower lobe (RLL), left upper lobe (LUL), and left lower lobe (LLL). H&E staining of lungs from a pig intubated with PVC-ETT. Extensive polymorphonuclear infiltration was found within the alveolar spaces, specifically, the arrow indicated polymorphonuclear cells (RUL). Infiltration of polymorphonuclear leukocytes, fibrinous exudates, and the arrows indicated cellular necrosis with disruption of cellular architecture (RML). The arrows indicated mucus plugs within the bronchiolar lumens, associated with bronchiolar wall alterations and surrounding inflammatory infiltration (RLL, LUL, LLL). Abnormal accumulations of red blood cells were observed in the alveoli (LUL). H&E staining of lungs from a pig intubated with CS-AgNPs@PAAm-Gelatin-ETT. All five lung lobes possessed intact alveolar walls with no inflammatory infiltration (RUL, RML, LUL, LLL). No mucous blockage was observed in the bronchioles (RLL). (100× scale bar = 100 μm, 400× scale bar = 50 μm).

## Data Availability

The authors declare that the main data supporting the findings of this study are available within the article and its Additional file, which are available from the corresponding authors upon reasonable request.

## Abbreviations

AAS: Atomic absorption spectrometry
AFM: Atomic force microscopy
AM: Acrylamide
ANOVA: One-way analysis of variance
AgNPs: Silver nanoparticles
BP: Benzophenone
BSA: Bovine serum albumin
CFU: Colony-forming units
CLSM: Confocal laser scanning microscopy
CS: Chitosan
EtOH: Ethanol
ETT: Endotracheal tube
FTIR: Fourier transform infrared
H&E: Hematoxylin and eosin
HAc: Acetic acid
HRTM: High-resolution transmission electron microscopy
HSA: Human serum albumin
IPN: Interpenetrating polymer network
LDH: Lactic dehydrogenase
LLL: Left lower lobe
LUL: Left upper lobe
PAAm: Polyacrylamide
PBS: Phosphate-buffered saline
PEEP: Positive end expiratory pressure
PI: Isoelectric points
PRP: Platelet-rich plasma
PVC: Polyvinyl chloride
QCM-D: Quartz crystal microbalance with dissipation monitoring
RLL: Right lower lobe
RML: Right middle lobe
RUL: Right upper lobe
SD: Standard deviation
SEM: Scanning electron microscopy
TA: Tannic acid
TSB: Tryptic soy broth
VAP: Ventilator-associated pneumonia
WCA: Water contact angle
XPS: X-ray photoelectron spectroscopy
XRD: X-ray diffraction
