# Supplementary material for: A novel antibacterial and antifouling nanocomposite coated endotracheal tube to prevent ventilator-associated pneumonia
Source: J Nanobiotechnology. 2022 Mar 5;20:112. doi: 10.1186/s12951-022-01323-x (PMC8897767; doi:10.1186/s12951-022-01323-x)
Supplement: Supplementary file 1 — Additional file 1: Figure S1. Fourier transform infrared spectra of PAAm-PVC. Figure S2. XRD spectra of the CS-AgNps@PAAm-Gelatin. Figure S3. Spacing of Ag (111) lattice fringe (d = 0.235 nm). Figure S4. The size distribution of CS-AgNPs. Figure S5. UV-visible spectra of silver-chitosan nanocomposite colloids prepared using different AgNO3 concentrations. Figure S6. Water contact angles of PVC. Figure S7. Stress-strain curves for the PVC-ETT (black) and CS-AgNps@PAAm-Gelatin-ETT (red) specimens. Figure S8. Standard process for the tensile test. Figure S9. General views of the commercial PVC-ETT and CS-AgNps@PAAm-Gelatin-ETT specimens. Observations of CS-AgNps@PAAm-Gelatin-ETT showed that the colorless coating made it indistinguishable from PVC-ETT, and no streaks or spots were observed on any surface. It was important that the cuff of the CS-AgNps@PAAm-Gelatin-ETT was not affected by the dip coating process, and could be inflated and deflated repeatedly to ensure its airtightness. Figure S10. Released amounts of Ag+ from CS-AgNPs@PAAm-Gelatin after immersion in PBS for up to 110 h. Figure S11. Zeta potential of PVC, PAAm-Gelation, and CS-AgNPs@PAAm-Gelatin. Figure S12. Porcine mechanical ventilation model with the oropharyngeal P. aeruginosa challenge. The tracheal endothelium was stained with hematoxylin and eosin (H&E). No sections exhibited inflammatory infiltration. (Scale bar = 100 μm). Figure S13. Porcine mechanical ventilation model with the oropharyngeal S. aureus challenge. The tracheal endothelium was stained with hematoxylin and eosin (H&E). No sections exhibited inflammatory infiltration. (Scale bar = 100 μm). Figure S14. Body temperature of pigs intubated with PVC-ETT and CS-AgNPs@PAAm-Gelatin-ETT respectively. Figure S15. Porcine mechanical ventilation model with the oropharyngeal S. aureus challenge. a Mucus adhesion to the ETTs after 48 h of mechanical ventilation. The PVC-ETT was clearly blocked by purulent secretions, while the CS-AgNPs@PAAm-Gelati [file 12951_2022_1323_MOESM1_ESM.docx]

**Supplementary Information**

**A novel antibacterial and antifouling nanocomposite coated endotracheal tube to prevent ventilator-associated pneumonia**

Yue Wang ^a^, Bingyue Cai ^b^, Dalong Ni ^c^, Yu Sun ^a^**^*^**, Gang Wang ^b^**^*^** and Hong Jiang ^a^**^*^**

^a^ Department of Anesthesiology, Shanghai Ninth People’s Hospital, Shanghai Jiao Tong University School of Medicine, No. 639 Zhizaoju Rd Shanghai 200011, P.R China

^b^ State Key Laboratory for Modification of Chemical Fibers and Polymer Materials, College of Materials Science & Engineering, Donghua University, No. 2999 Renmin Rd, Shanghai 201620, P.R China

^c^ Department of Orthopaedics, Shanghai Key Laboratory for Prevention and Treatment of Bone and Joint Diseases, Shanghai Institute of Traumatology and Orthopaedics, Ruijin Hospital, Shanghai Jiao Tong University School of Medicine, No. 197 Ruijin 2nd Rd, Shanghai 200025, P.R China

**^*^** Corresponding author

E-mail: [SUNY1333@sh9hospital.org.cn](mailto:SUNY1333@sh9hospital.org.cn) (Y. Sun), [gwf8707@dhu.edu.cn](mailto:gwf8707@dhu.edu.cn) (G. Wang), dr_jianghong@163.com (H. Jiang)

Yue Wang and Bingyue Cai contributed equally to this work.

**Additional Figures**

**
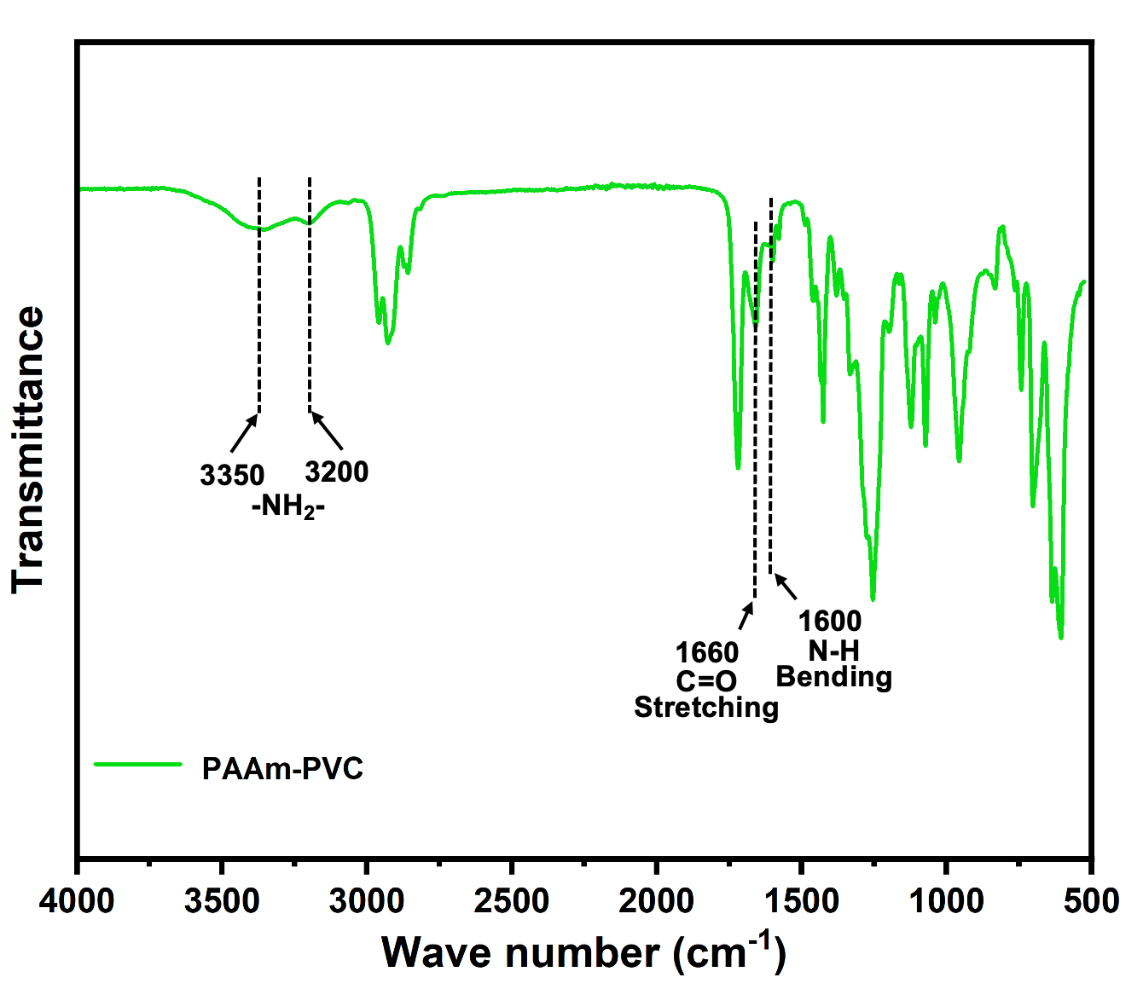
**

**Figure S1:** Fourier transform infrared spectra of PAAm-PVC.


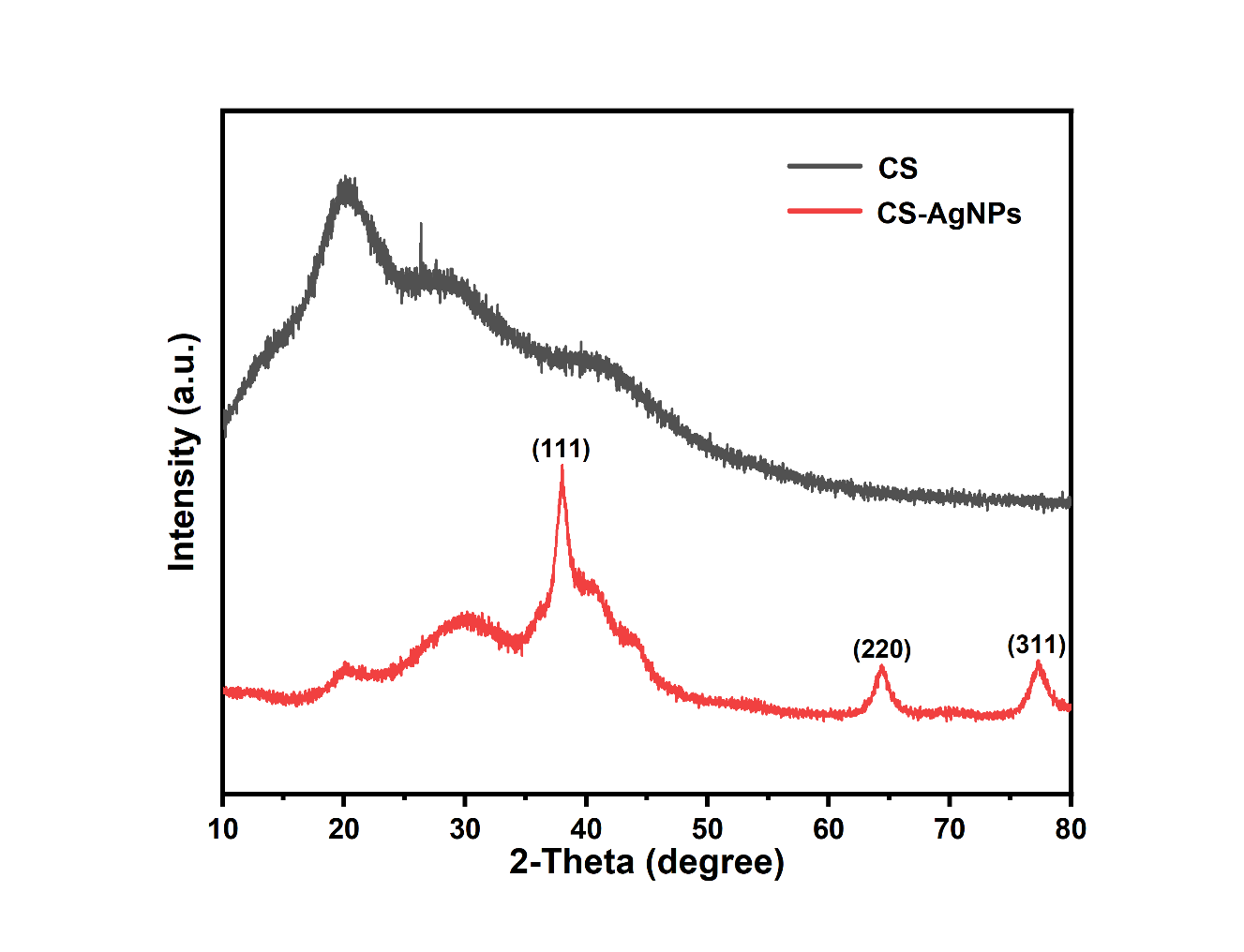


**Figure S2**. XRD spectra of the CS-AgNps@PAAm-Gelatin.

**
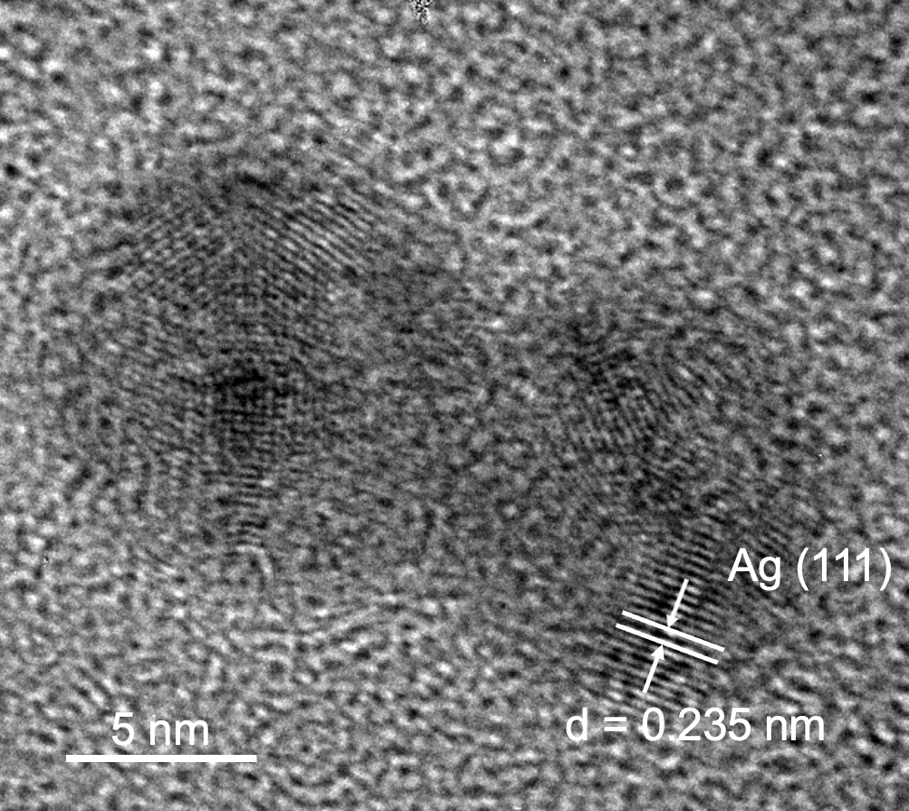
**

**Figure S3**. Spacing of Ag (111) lattice fringe (d = 0.235 nm).

**
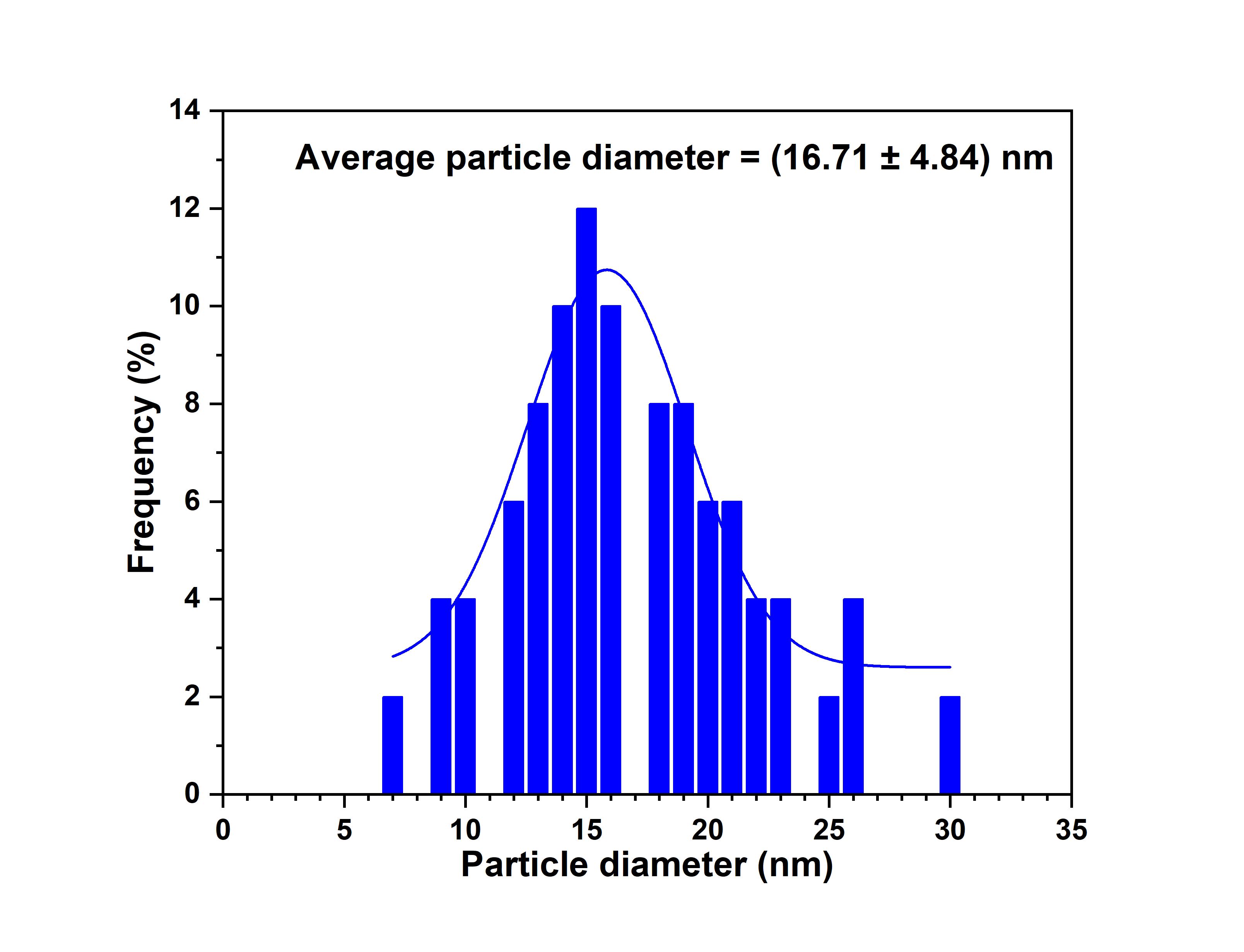
**

**Figure S4**. The size distribution of CS-AgNPs.


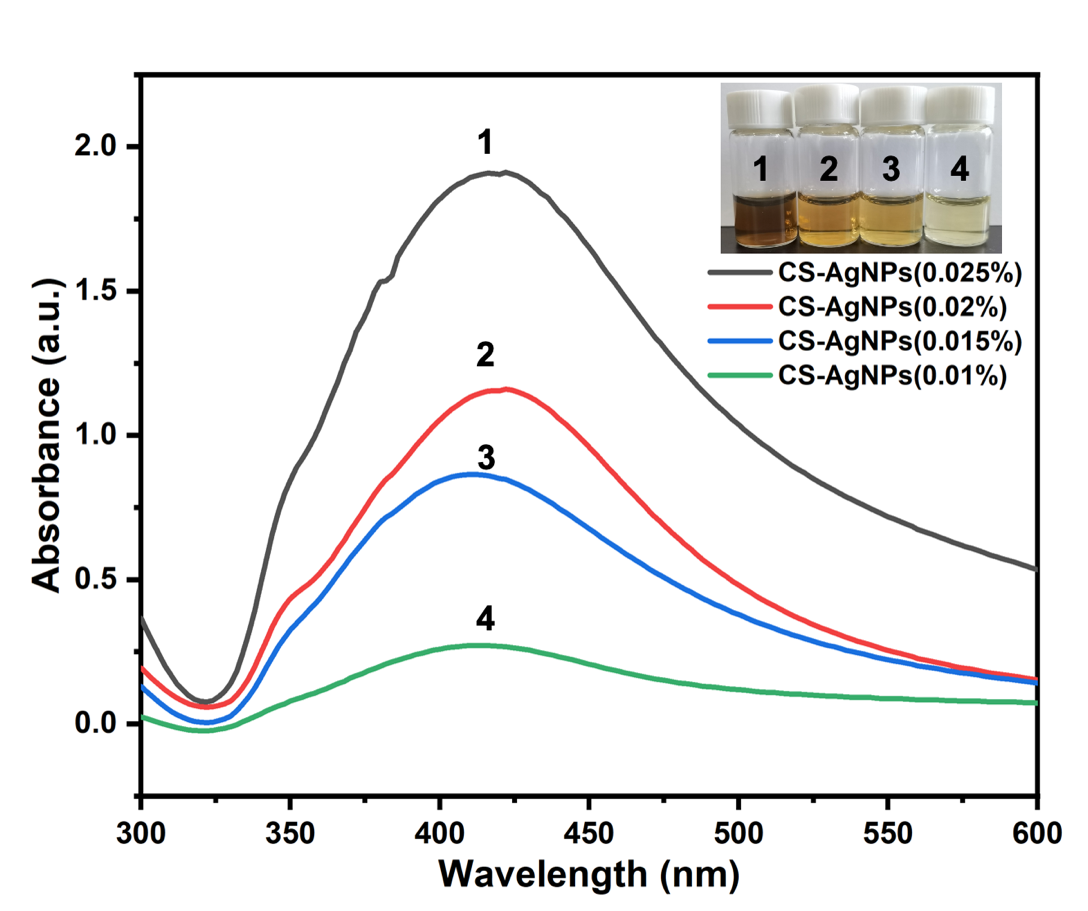


**Figure S5**. UV-visible spectra of silver-chitosan nanocomposite colloids prepared using different AgNO_3_ concentrations.

**
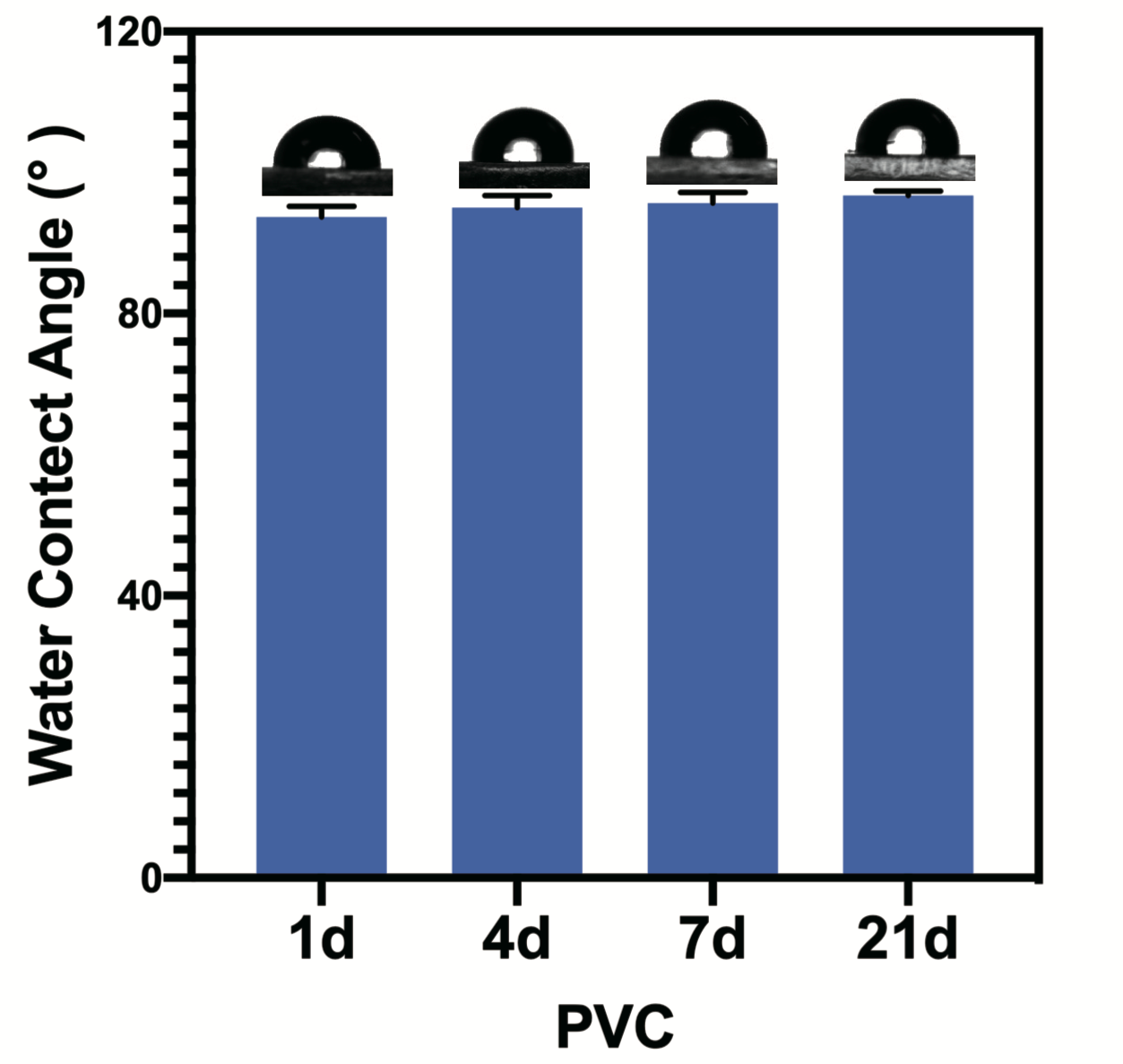
**

**Figure S6**. Water contact angles of PVC**.**

**
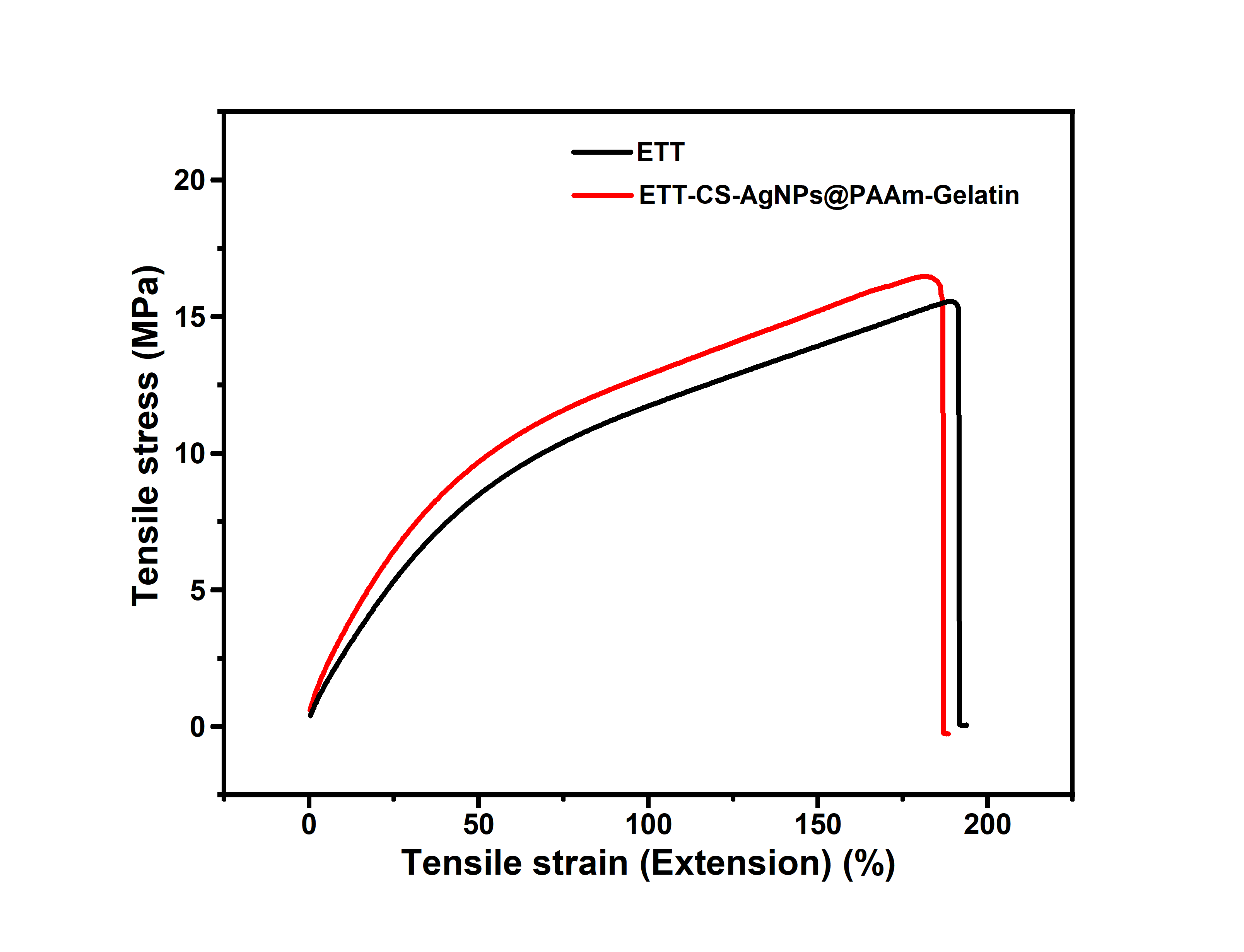
**

**Figure S7**. Stress-strain curves for the PVC-ETT (black) and CS-AgNps@PAAm-Gelatin-ETT (red) specimens.


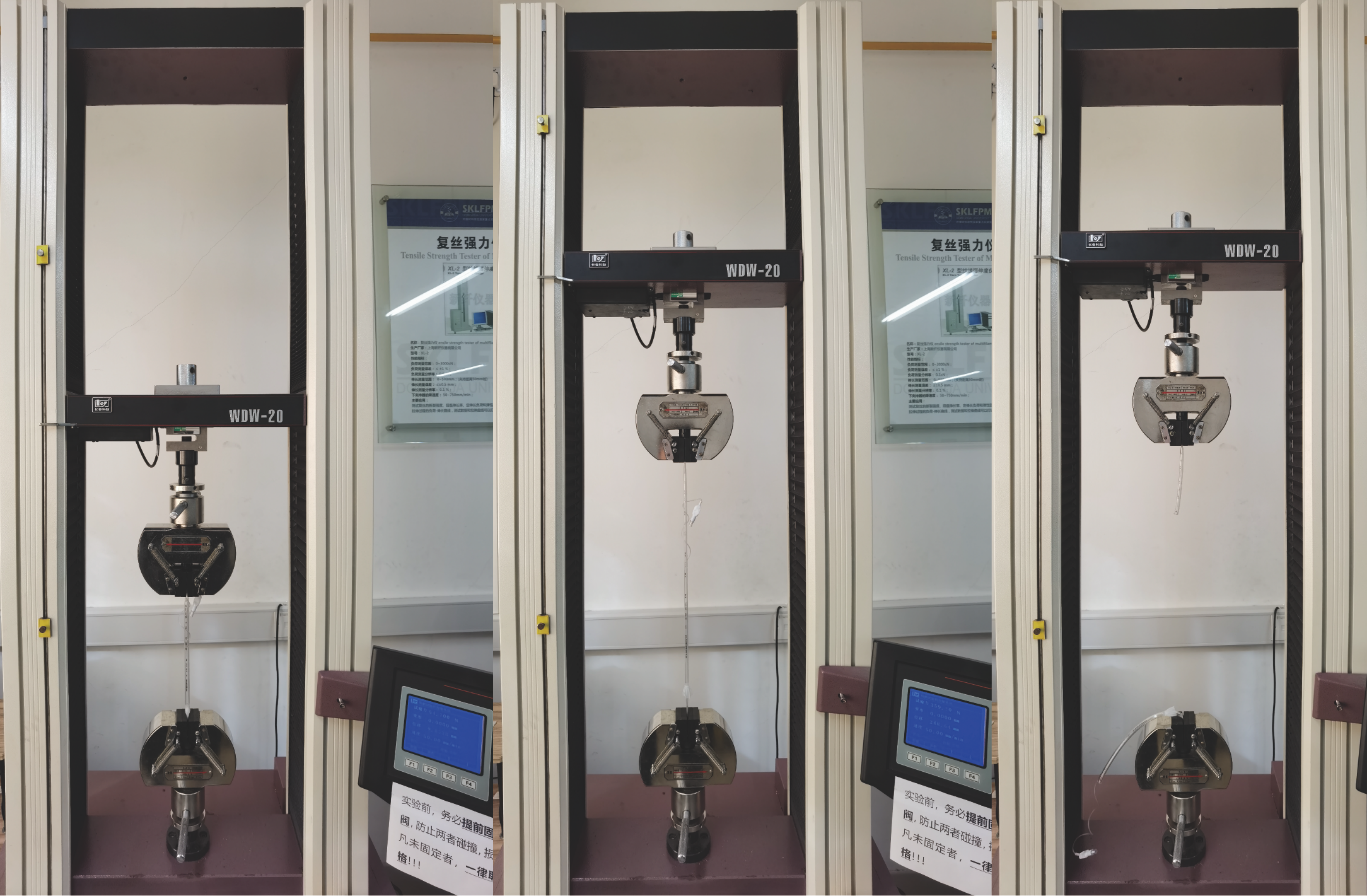


**Figure S8**. Standard process for the tensile test.


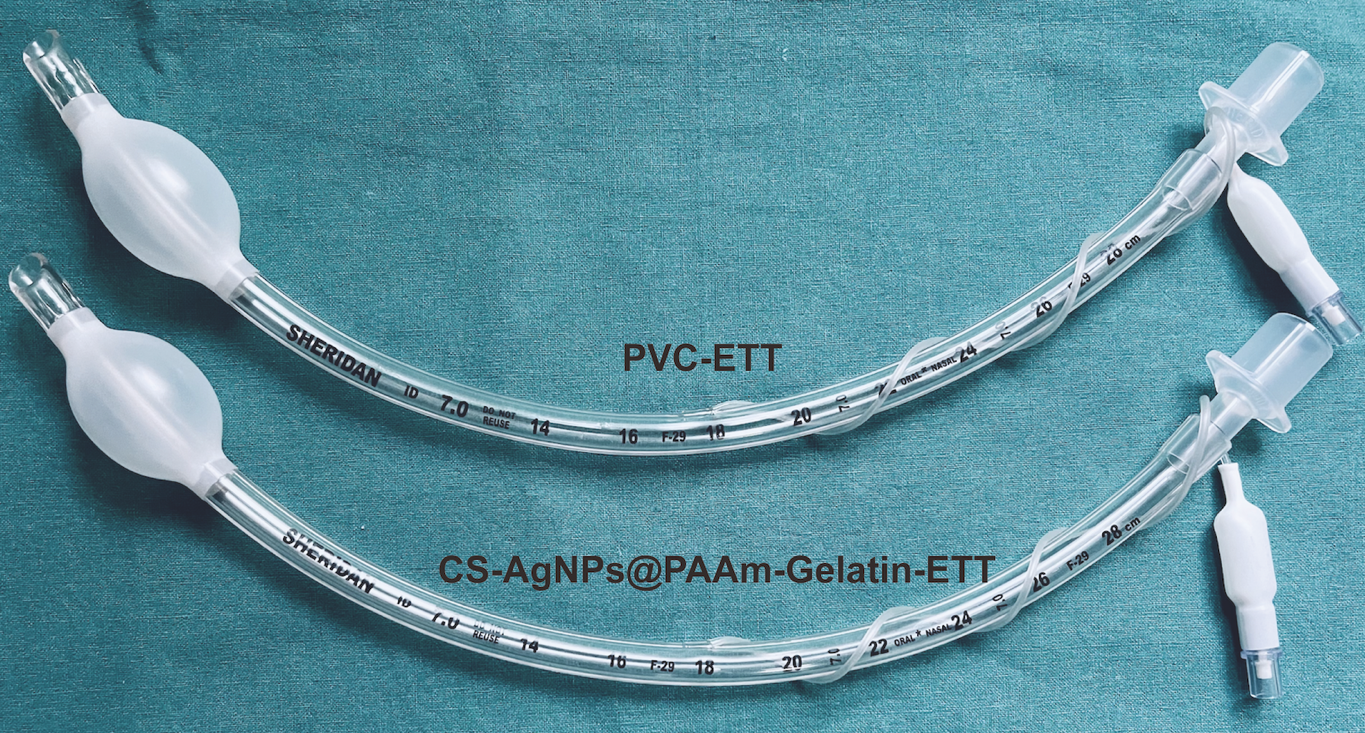


**Figure S9.** General views of the commercial PVC-ETT and CS-AgNps@PAAm-Gelatin-ETT specimens. Observations of CS-AgNps@PAAm-Gelatin-ETT showed that the colorless coating made it indistinguishable from PVC-ETT, and no streaks or spots were observed on any surface. It was important that the cuff of the CS-AgNps@PAAm-Gelatin-ETT was not affected by the dip coating process, and could be inflated and deflated repeatedly to ensure its airtightness.

**
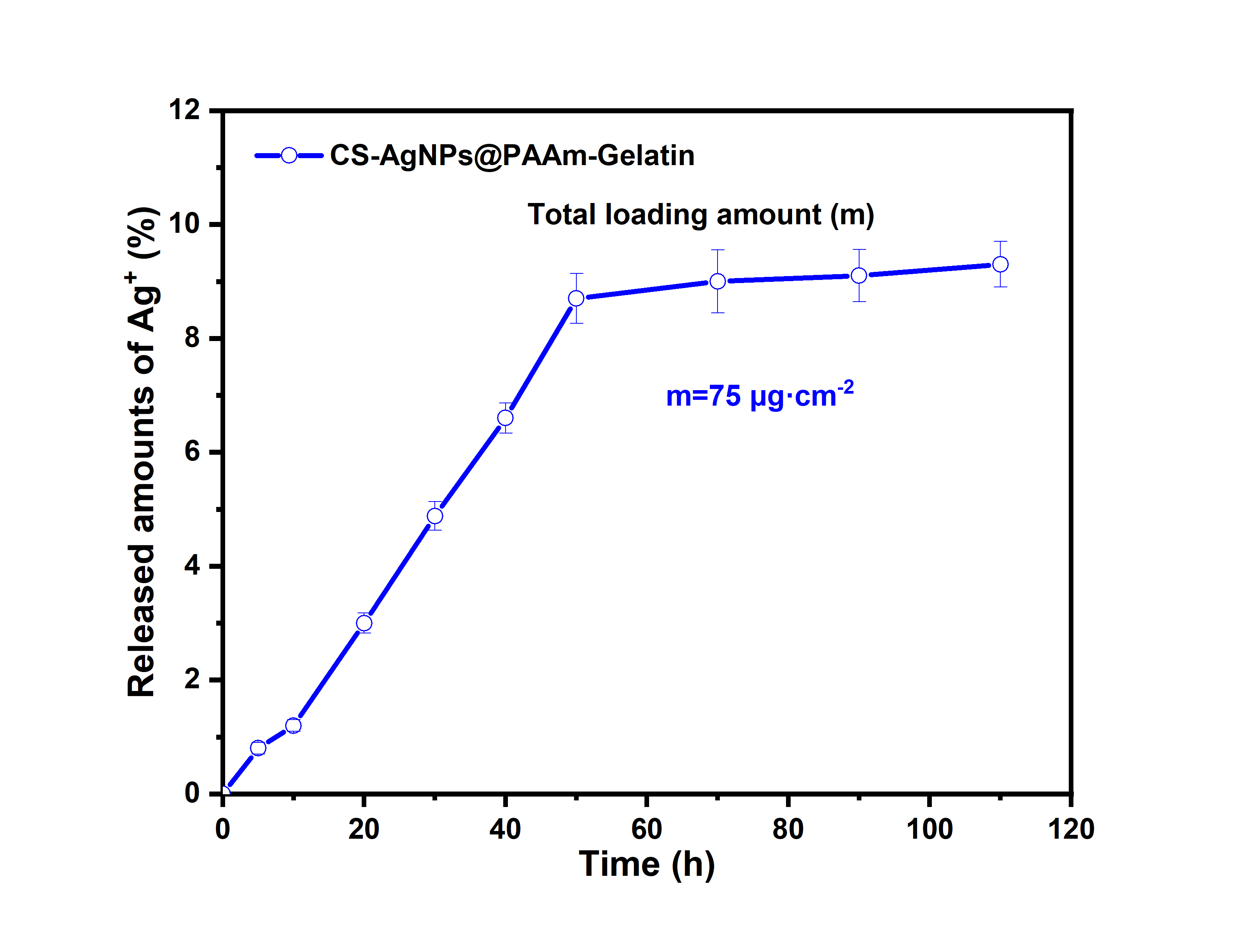
**

**Figure S10**. Released amounts of Ag^+^ from CS-AgNPs@PAAm-Gelatin after immersion in PBS for up to 110 h.


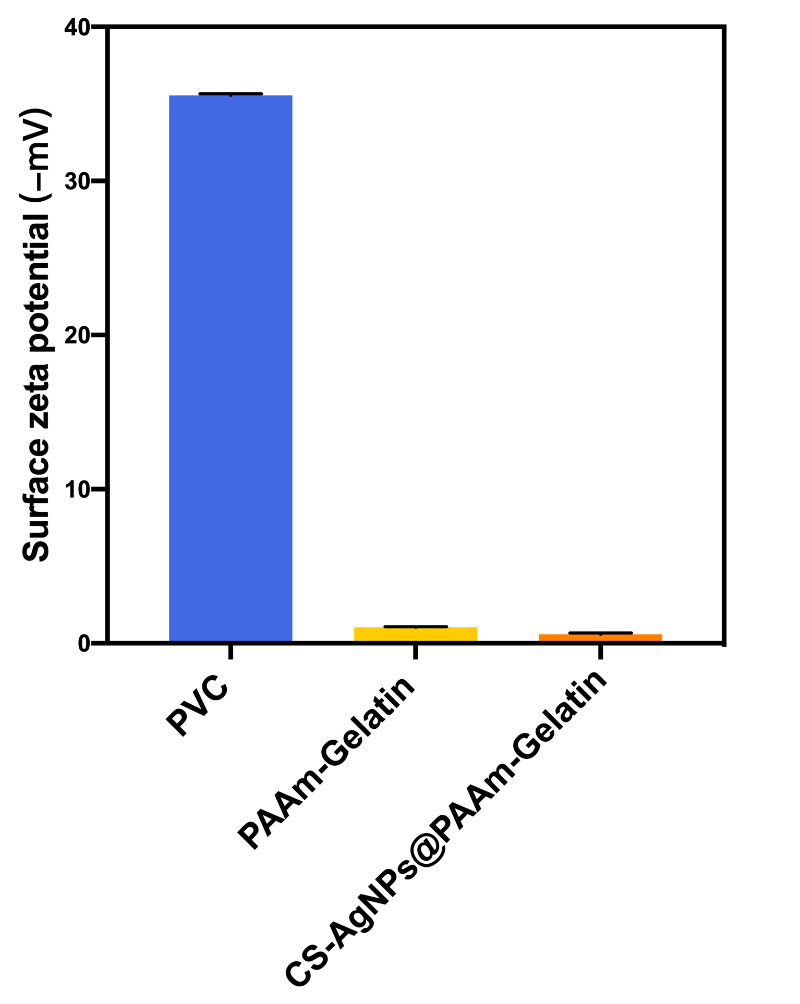


**Figure S11.** Zeta potential of PVC, PAAm-Gelation, and CS-AgNPs@PAAm-Gelatin.


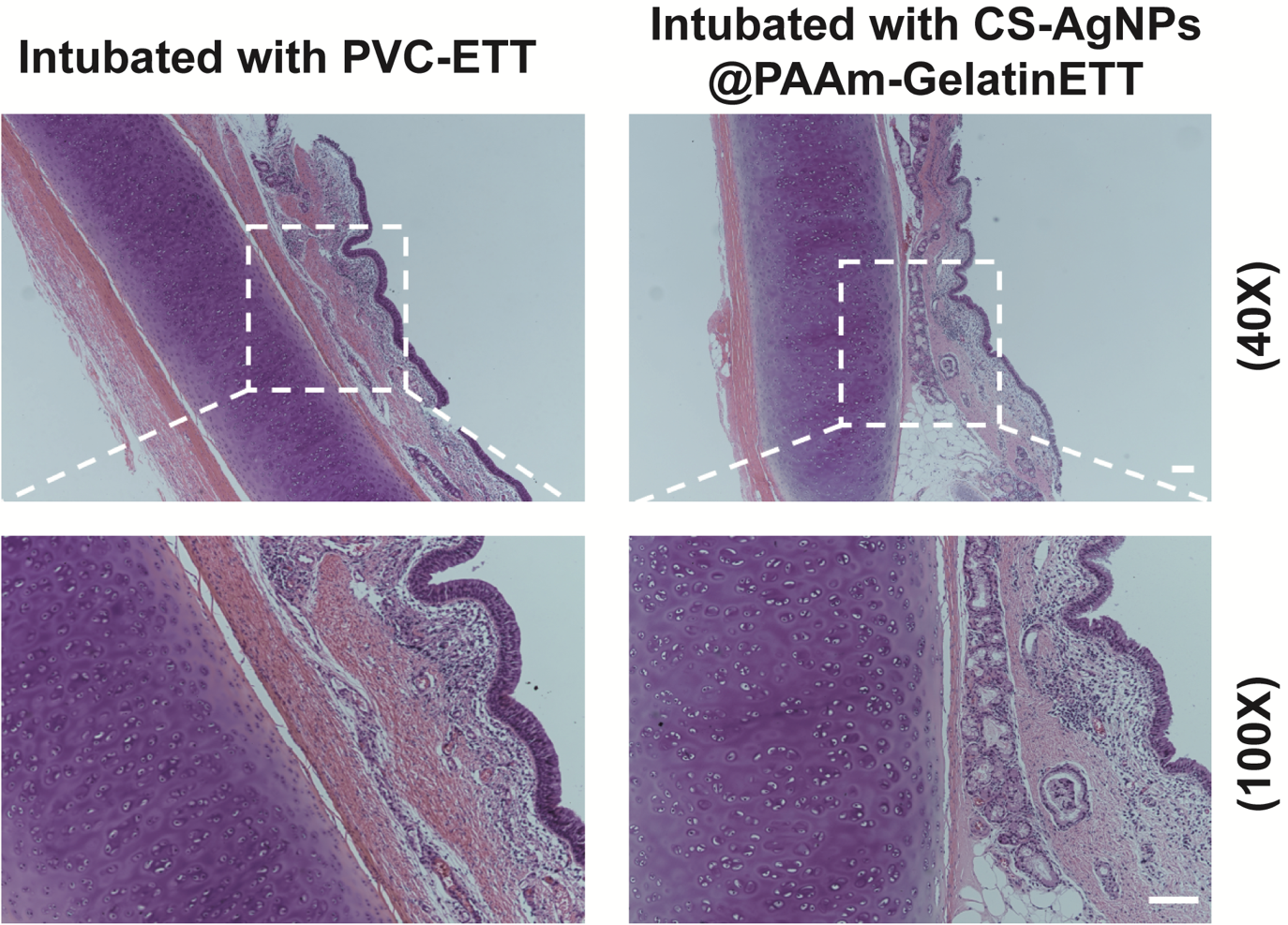


**Figure S12.** Porcine mechanical ventilation model with the oropharyngeal *P. aeruginosa* challenge. The tracheal endothelium was stained with hematoxylin and eosin (H&E). No sections exhibited inflammatory infiltration. (Scale bar = 100 μm)


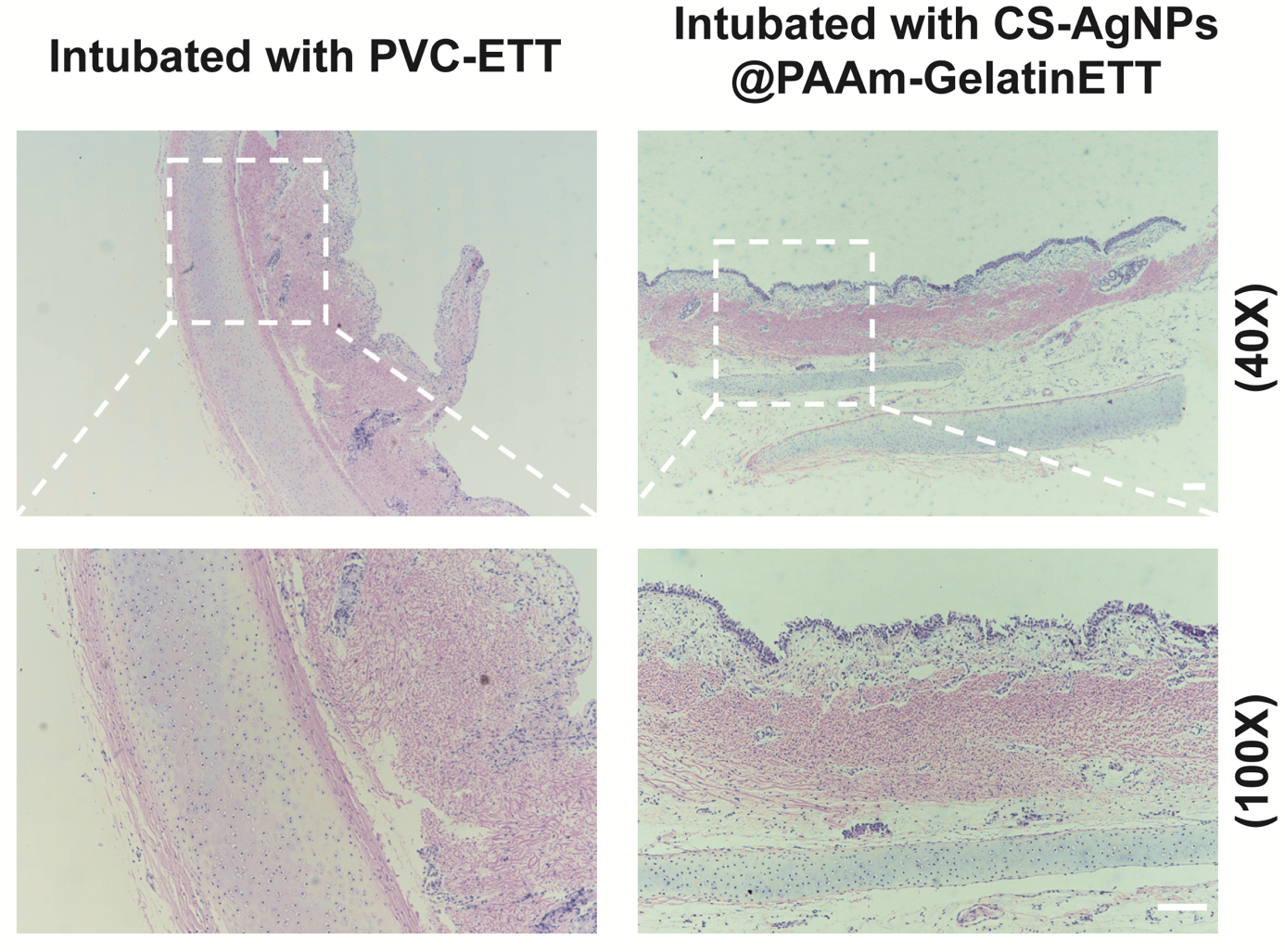


**Figure S13.** Porcine mechanical ventilation model with the oropharyngeal S. aureus challenge. The tracheal endothelium was stained with hematoxylin and eosin (H&E). No sections exhibited inflammatory infiltration. (Scale bar = 100 μm)


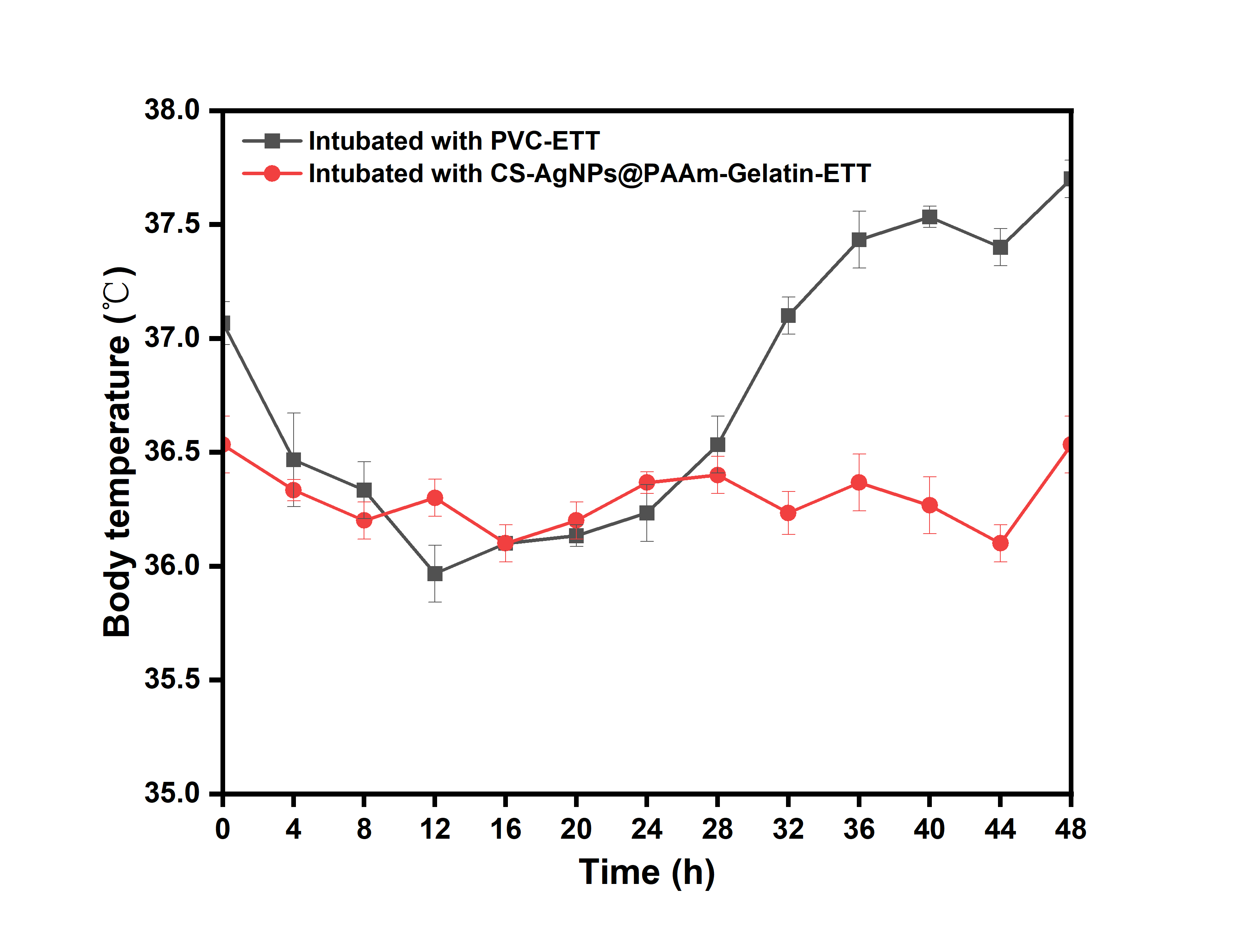


**Figure S14.** Body temperature of pigs intubated with PVC-ETT and CS-AgNPs@PAAm-Gelatin-ETT respectively.

**
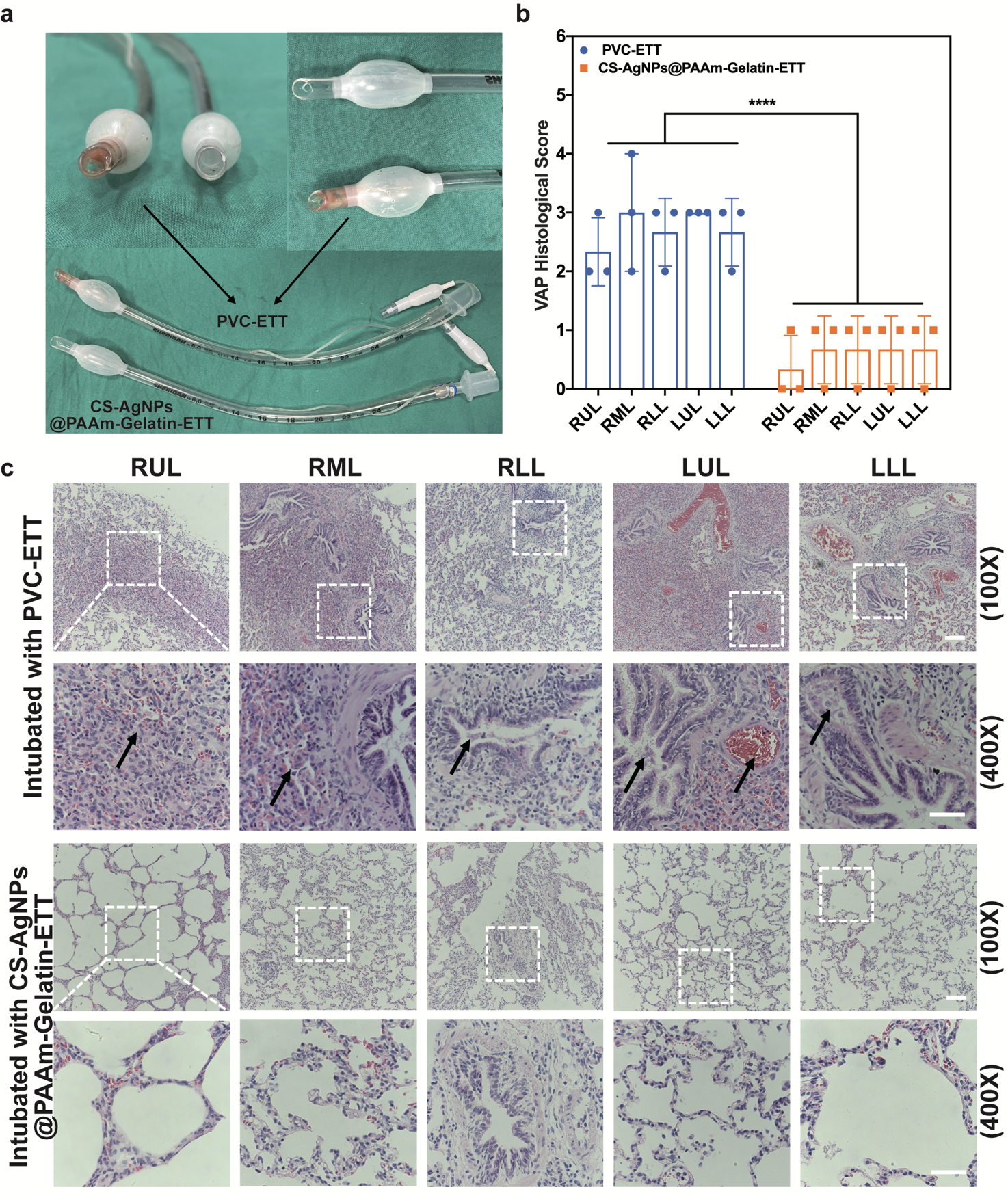
**

**Figure S15**. Porcine mechanical ventilation model with the oropharyngeal *S. aureus* challenge. a. Mucus adhesion to the ETTs after 48 h of mechanical ventilation. The PVC-ETT was clearly blocked by purulent secretions, while the CS-AgNPs@PAAm-Gelatin-ETT was clean and unobstructed. b. VAP histological score of pigs intubated with PVC-ETTs and CS-AgNPs@PAAm-Gelatin-ETTs: 0, no injury; 1, purulent mucous plugging; 2, bronchiolitis; 3, pneumonia; 4, confluent pneumonia; and 5, abscessed pneumonia (****p < 0.0001). c. All pulmonary sections were stained with hematoxylin and eosin (H&E). Right upper lobe (RUL), right middle lobe (RML), right lower lobe (RLL), left upper lobe (LUL), and left lower lobe (LLL). H&E staining of lungs from a pig intubated with PVC-ETT. Extensive polymorphonuclear infiltration was found within the alveolar spaces, specifically, the arrow indicated polymorphonuclear cells (RUL). Infiltration of polymorphonuclear leukocytes, fibrinous exudates, and the arrows indicated cellular necrosis with disruption of cellular architecture (RML). The arrows indicated mucus plugs within the bronchiolar lumens, associated with bronchiolar wall alterations and surrounding inflammatory infiltration (RLL, LUL, LLL). Abnormal accumulations of red blood cells were observed in the alveoli (LUL). H&E staining of lungs from a pig intubated with CS-AgNPs@PAAm-Gelatin-ETT. All five lung lobes possessed intact alveolar walls with no inflammatory infiltration (RUL, RML, LUL, LLL). No mucous blockage was observed in the bronchioles (RLL). (100× scale bar = 100 μm, 400× scale bar = 50 μm)
